# An unrecognized mechanism of self-protection in degenerating neurons mediated by astrocytic YAP through Wnts/β-catenin/EAAT2 signaling in C9orf72-poly-GA mice

**DOI:** 10.7150/thno.113599

**Published:** 2025-07-24

**Authors:** Dongmei Li, Yan Wei, Rui Yang, Xuan Luo, Yanzhu Liu, Weiqiao Zhao, Hui Yang, Yumin Wu, Ying Wang, Zhihui Huang

**Affiliations:** 1 School of Pharmacy, Hangzhou Normal University, Hangzhou 311121, Zhejiang, China.; 2 Department of Clinical Research Center, Affiliated Hangzhou First People's Hospital, Westlake University School of Medicine, Hangzhou 310006, Zhejiang, China.; 3 School of Basic Medicine, Hangzhou Normal University, Hangzhou 311121, Zhejiang, China.; 4 Department of Orthopedics (Spine Surgery), the First Affiliated Hospital of Wenzhou Medical University, Wenzhou 325000, Zhejiang, China.

**Keywords:** YAP, ALS, EAAT2, neurodegeneration, motor dysfunction

## Abstract

**Rationale:** Amyotrophic lateral sclerosis (ALS) is a fatal neurodegenerative disorder characterized by the progressive loss of motor neurons in the central nervous system (CNS). Non-neuronal cells, particularly astrocytes, have been recognized as pivotal contributors to ALS onset and progression. However, the underlying mechanisms of interactions between astrocytes and motor neurons during ALS remain unclear. Recent studies have identified the neuronal Hippo kinase mammalian sterile 20-like kinase 1 (MST1) as a key regulator of neurodegeneration in ALS. Yes-associated protein (YAP), a major downstream effector of the Hippo pathway, is predominantly expressed in astrocytes. However, the role of astrocytic YAP in ALS and its underlying mechanisms remain unexplored.

**Methods:** To evaluate the function of YAP in ALS, we established a C9orf72-poly-GA mouse model (ALS mice) via intracerebroventricular injection of AAV viruses. Furthermore, mice with conditional knockout (CKO) of YAP in astrocytes (YAP^GFAP^-CKO mice) were generated and then YAP^GFAP^-CKO ALS mice and their littermate controls (YAP^f/f^ ALS mice) were used as experimental subjects. Behavioral tests, immunostaining, Nissl staining, quantitative real-time PCR (qPCR), and Western blotting were used to assess the effects of astrocytic YAP deletion in ALS progression. In addition, we investigated the role and mechanism of astrocytic YAP in the pathogenesis of ALS by integrating RNA sequencing (RNA-seq) from primary cultured astrocytes with single-nucleus transcriptomic (snRNA-seq) from C9orf72-ALS/FTD patients. Then, *in vitro* experiments including primary cultured astrocytes and neurons were used to further elucidate the potential molecular mechanism of astrocytic YAP in ALS. Finally, we evaluated the therapeutic effects of the excitatory amino acid transporter-2 (EAAT2) activator LDN-212320 and the Hippo kinase MST1/2 inhibitor XMU-MP-1 as candidate treatments for ALS.

**Results:** We found that YAP was upregulated and activated specifically in astrocytes, but not in neurons or microglia, within the motor cortex of ALS mice. Conditional knockout of YAP in astrocytes exacerbated motor deficits, neuronal loss, pathological translocation of TDP-43, inflammatory infiltration, and reduced astrocytic proliferation in ALS mice. Mechanistically, Wnts secreted by degenerating neurons and astrocytes activated YAP/β-catenin signaling and further promoted the expression of EAAT2 in astrocytes, which prevented neuronal glutamate excitotoxicity, neuronal loss, and motor dysfunction in ALS mice. Interestingly, treatment with LDN-212320 promoted EAAT2 expression and partially restored motor deficits and neuronal loss in YAP^GFAP^-CKO ALS mice. Finally, activation of YAP by XMU-MP-1 upregulated β-catenin and EAAT2 expression, and partially alleviated motor deficits and neurodegeneration in ALS mice.

**Conclusions:** These results identify an unrecognized mechanism of self-protection in degenerating neurons mediated by astrocytic YAP through Wnts/β-catenin/EAAT2 signaling to prevent glutamate excitotoxicity of neurons in ALS mice, and provide a novel drug target for ALS.

## Introduction

Amyotrophic lateral sclerosis (ALS) is a fatal neurodegenerative disorder characterized by the progressive degeneration of motor neurons in the brain and spinal cord leading to muscle weakness, eventual paralysis, and death from respiratory paralysis within 3 to 5 years 1-3. Despite decades of intensive research, there is no cure for ALS. Currently, three pharmaceutical compounds with an effect on disease progression are approved: Riluzole (a glutamate antagonist), Edaravone (a free radical scavenger) and Sodium phenylbutyrate/Taurursodiol 4. In addition, a combination of dextromethorphan hydrobromide and quinidine sulfate (Nuedexta) is used for the treatment of pseudobulbar affect (PBA) in ALS pathogenesis 5. Globally, efforts are underway to prevent or alleviate the symptoms of this neurodegenerative disease, including the implementation of antisense oligonucleotides (ASOs), induced pluripotent stem cells (iPSCs), CRISPR-Cas9 technology, non-invasive brain stimulation or ALS-on-a-chip technology 6. However, the treatment of ALS is largely limited to palliative care, so it is urgent to develop new and effective disease improvement therapies.

ALS is affected by many pathological mechanisms such as glutamate-induced excitotoxicity, apoptosis, mitochondrial dysfunction, oxidative stress, protein misfolding, neuroinflammation, altered RNA metabolism and neurofilament abnormalities 7-9. Approximately 90~95% of ALS cases are sporadic (sALS), with no obvious genetic link, while the remaining 5~10% are familial (fALS) 10. Many important gene mutations have been identified in fALS, including mutations in Cu/Zn superoxide dismutase 1 gene (*SOD1*), Chromosome 9 Open Reading Frame 72 (*C9ORF72*), TAR DNA Binding Protein (*TARDBP*), Fused in Sarcoma (*FUS*) gene and TANK-binding kinase 1 (*TBK1*) 11. The above studies mainly focus on the pathogenic mechanisms of motor neuron degeneration in ALS, however, non-neuronal cells such as astrocytes have been recognized to play a pivotal role in disease onset and progression of ALS 12-14. Astrocytes have critical roles in adult central nervous system (CNS) homeostasis, including synaptic glutamate uptake and synapse plasticity, maintenance of extracellular potassium and nutrient support of neurons, and regulation of blood brain barrier, water flux, ion and pH homeostasis 15. Initial evidence for astrocytic contribution to motor neuron damage and ALS progression came from studies of decrease and dysfunction of astrocyte glutamate transporter GLT1 (EAAT2 in human) in ALS patients or ALS animal models 16, 17. The decrease and dysfunction of EAAT2 in astrocytes is the main cause of glutamate-mediated excitotoxicity in ALS 18. Furthermore, transplantation of wild-type lineage-restricted astrocyte precursors around the cervical spinal cord of SOD1^G93A^ rats slowed the progression of ALS and prolonged survival probability 19. Oppositely, transplantation of astrocyte precursors carrying SOD1^G93A^ mutation induced the local degeneration of motor neurons in wild-type spinal cord 20. Nowadays, although several mechanisms of astrocytic contribution to ALS pathology and progression have been addressed, including loss of support mechanisms and toxic gain of function mechanisms 14, the detailed molecular mechanisms of interactions between astrocytes and motor neurons during ALS remain unclear.

The Hippo pathway is critical in organ size and tissue homeostasis, and its dysregulation has been implicated in several diseases including tumorigenesis, cardiac diseases, pulmonary diseases, renal diseases and immune dysfunction 21. The core of the Hippo pathway, including mammalian sterile 20-like kinases 1/2 (MST1/2), salvador homolog 1 (SAV1), Mps one binder 1A and B (MOB1A/B), large tumor suppressor 1/2 (LATS1/2), Yes-associated protein (YAP) and transcriptional co-activator with PDZ binding motif (TAZ), constitutes a kinase cascade reaction, with YAP acting as a major downstream effector of the Hippo pathway 21, 22. After the Hippo signal is activated, the MST1/2 and SAV1 phosphorylate and activate the downstream kinase LATS1/2, and then LATS1/2 and MOB1A/B phosphorylate the transcriptional co-activators YAP and TAZ, leading to degradation or retention of YAP in the cytoplasm 23, 24. When the Hippo signaling is inhibited, MST1/2 and LATS1/2 are deactivated, which promotes YAP to accumulate in the nucleus and combine with the transcriptional enhanced associate domain (TEAD) family, and finally induces the expression of genes 25. Interestingly, motor neurons in sALS patients and SOD1^G93A^ mice exhibit the elevated MST1 activity, and MST1 knockout delays the onset of disease and prolongs the survival of SOD1^G93A^ mice 26. In addition, loss of function of Hippo (Drosophila MST) rescues the deficits in the FUS-ALS Drosophila model 27. Another study has also clarified that inactivation of Hippo signaling and JNK signaling alleviates FUS-mediated neurodegeneration in Drosophila eyes 28. Collectively, these studies demonstrate that neuronal MST1/Hippo in a YAP-independent manner contributes to neurodegeneration in ALS. YAP is abundant in astrocytes and plays a critical role in regulating astrocytic proliferation and differentiation 29-32. Growing evidence has revealed the function of astrocytic YAP under pathological conditions, such as promoting the formation of glial scars and neural regeneration of mice after spinal cord injury 33, promoting cholesterol synthesis in experimental autoimmune encephalomyelitis (EAE) to prevent demyelination 34, and upregulating TGF-β signaling to prevent neuroinflammatory infiltration and demyelination in multiple sclerosis-related optic neuritis (MS-ON) 35. However, the roles and mechanisms of astrocytic YAP signaling in ALS remain poorly understood.

Wnt signaling is mediated through β-catenin dependent pathway, also known as the canonical pathway (Wnt/β-catenin pathway) and β-catenin independent pathway, referred to as the noncanonical pathway (Wnt/Ca^2+^ and Wnt/planar cell polarity) 36. Canonical pathway is very important for neurogenesis, cell proliferation and neurogenesis, while noncanonical pathway is responsible for cell polarity, calcium maintenance and cell migration 37. Recent studies have shown that Wnts, their receptors, and core components of the Wnt signaling pathway are expressed in ALS transgenic mice and ALS patients 38. The activation of Wnt signaling pathway was identified in hiPSC and mouse ALS astrocytes 39. Abnormal activation of the Wnt/β-catenin signaling pathway is related to neuronal degeneration and glial cell proliferation in ALS 40. In addition, there are hypotheses proposing that the activation of the Wnt/β-catenin pathway in astrocytes could be a neuroprotective attempt to counteract motor neuron pathology in ALS 41. Interestingly, the crosstalk between Hippo-YAP/TAZ and Wnt/β-catenin signaling pathways has been variously inferred in different physiological and pathophysiological conditions 42-44. For example, YAP/TAZ as critical mediators of alternative Wnt signaling regulates its biological responses 45, and Wnt/β-catenin signaling regulates YAP/TAZ activity during embryonic development in zebrafish 46. Also, Wnt/β-catenin pathway has been defined as an essential signaling system not only for neurodevelopment, but especially for the survival, neuroprotection and self-repair against neurodegeneration, inflammation and oxidative stress, via astrocyte-microglial-neuron crosstalks mediated by Wnts 47-50. However, the potential crosstalk between Hippo-YAP/TAZ and Wnt/β-catenin signaling pathways in ALS, and its contribution to ALS remain unclear.

In this study, we established a C9orf72-poly-GA mouse model (ALS mice) to investigate the function of astrocytic YAP in ALS. We found that YAP was upregulated and activated in astrocytes, but not in neurons or microglia, within the motor cortex of ALS mice, and the knockout of astrocytic YAP exacerbated the disease progression of ALS. Mechanistically, we found that degenerating neurons and astrocytes secreted Wnts, which activated YAP/β-catenin signaling and further promoted EAAT2 expression in astrocytes, thereby preventing neuronal glutamate excitotoxicity, neuronal loss, and motor dysfunction in ALS mice. Our study reveals an unrecognized function of astrocytic YAP in preventing glutamate excitotoxicity of neurons through Wnts/β-catenin/EAAT2 signaling in ALS mice, and provide a new drug target for ALS.

## Materials and Methods

### Animals

YAP^GFAP^-CKO mice were generated by crossing floxed YAP allele (YAP^f/f^) with GFAP-Cre mice (from the Jackson Laboratory), as described previously 35. Both YAP^f/f^ and YAP^GFAP^-CKO mice were maintained under the background of C57BL/6 strain and these mice were genotyped. The use of animals and all relevant experimental protocols have acquired the approval of the Animal Care and Use Committee of Hangzhou Normal University (HSD20220911).

### Intracerebroventricular injections

C9orf72-poly-GA mice were described previously 51. Briefly, pups from YAP^f/f^ and YAP^GFAP^-CKO mice were cryoanesthetized on ice at postnatal day 0 (P0). Then, a 32-gauge Hamilton needle was attached to a 10 µl Hamilton syringe and inserted vertically at two-fifths of the distance between the pup's eyes and lambda. The vertical depth of that needle was kept at 2 mm. 4 µl (2 µl per side) of AAV8 viruses (AAV-GFP-GA5, 6.60 × 10¹³ vg/ml; AAV-GFP-GA100, 4.11 × 10¹³ vg/ml; WZ Biosciences Inc, viral sequences gifted from Prof. Chengyong Shen, Zhejiang University) were slowly injected into the cerebral ventricle. After the injection, the pups were placed on a heating pad until they recovered from anesthesia and then were returned to their home cages. Behavioral analyses were conducted when the mice reached 3 months of age.

### Behavioral analysis

A series of behavioral tests were conducted on 3-month-old YAP^f/f^ ALS mice, YAP^GFAP^-CKO ALS mice, and their littermate YAP^f/f^ and YAP^GFAP^-CKO control mice to evaluate motor function 52. All mice were acclimated to the laboratory for at least 1 h before the formal experiment and were returned to their home cages after each experiment. No more than two tests were performed each day. The testing room was kept quietly and forbidden to disturb the spontaneous activity of mice.

### Tail-suspension test

Briefly, 3-month-old YAP^f/f^ and YAP^GFAP^-CKO control mice, YAP^f/f^ ALS and YAP^GFAP^-CKO ALS mice were placed in a tail-suspension tester. The duration of hind limb clasping was recorded for 3 min.

### Cage behavior test

Cage behavior test was an experiment to test the movement balance and coordination ability of mice. Briefly, the mice were put on the edge of the cage to walk on the premise that should never be subjected to this test before. The motor performance of mice was recorded with in 3 min.

### Rotarod test

The exercise ability of mice was evaluated using the rotarod test. Briefly, one day before the test, the mice were placed on the rod for 2 min at a constant speed of 4 rpm to adapt and train. On the test day, the rod was set to accelerate from 4 rpm to 40 rpm over 5 min, and the latency to fall was recorded for each mouse. The test was repeated three times for each mouse with an interval of 15 min.

### Balance beam test

The balance beam test was used to test the balance ability, muscles and coordination of mice. On the first day, each mouse was trained to run across the beam without interruption. On the second day, the camera was used to record the process of each mouse crossing the beam and the number of times each mouse's hind limbs slipped.

### Footprint test

The footprint test was used to assess the motor function of the forelimbs and hind limbs. Briefly, the mice ran successfully along the paper track during training. In the formal experiment, the fore paws and hind paws of mice were coated with red and blue dyes, respectively. The gait of the mice was recorded. The center of each paw was marked, and the distance between the fore paw and hind paw at the same side was measured as back-front distance, and the distance between hind paws at the same side was measured as back stride distance.

### Immunostaining

The cultured cells were washed with phosphate buffered saline (PBS) three times, fixed with 4% paraformaldehyde (PFA) for 20 min, and then permeabilized and blocked in PBS containing 0.1% Triton X-100 (#T8200, Solarbio) and 5% bovine serum albumin (BSA, #V900933, Sigma-Aldrich) for 1 h. The cells were subsequently incubated overnight at 4℃ with primary antibody, including rat anti-EAAT2 (1:200, OB-PRT026, Oasis Biofarm), mouse anti-GFAP (1:500, MAB360, Millipore), rabbit anti-YAP (1:100, #14074, CST), and rabbit anti-MAP2 (1:400, #4542, CST). The cells were washed three times with PBS (5 min each time), and then incubated with appropriate secondary antibodies [(1: 1, 000, Invitrogen Alexa Fluor™ :Plus 488 goat anti-rabbit IgG (H+L), #A32731; 546 donkey anti-rabbit IgG (H+L), #A11040; 546 goat anti-mouse IgG (H+L), #A11030; 1:1, 000, Oasis Biofarm: 647 goat anti-Rat IgG (H+L), #G-RT647; 1: 1, 000, Beyotime Alexa Fluor: 488 goat anti-mouse IgG (H+L), #A0428)] and DAPI for labeling of nuclei (1:1, 000, #4083S, CST) in 5% BSA at room temperature incubation for 1 h. After PBS washing, the cells were mounted. Image acquisition was done by Nikon single-photon and two-photon integrated confocal microscope (Nikon AX R MP), and analyzed by the ImageJ software.

For tissue section staining, mice were perfused with PBS followed by 4% PFA through the heart. The brains were then extracted and immersed in 4% PFA for 24-48 h. After fixation, the brains were transferred to 15% and 30% sucrose solution for continuous dehydration for 24-48 h. Subsequently, 20 μm sections were cut using a cryosectioner (CryoStar NX50, Thermo Scientific). The brain tissue sections were stained, fixed for 30 min, and then repaired with sodium citrate antigen repair solution at 65℃ for 45 min, permeabilized and blocked with 5% BSA (#V900933, Sigma-Aldrich) containing 0.3% Triton X-100 (#T8200, Solarbio) at room temperature for 1.5 h, followed by overnight incubation with primary antibodies at 4℃. The next day, the sections were washed three times and incubated with the appropriate secondary antibody [(1: 1, 000, Invitrogen Alexa Fluor™ :Plus 488 goat anti-rabbit IgG (H+L), #A32731; 546 donkey anti-rabbit IgG (H+L), #A11040; 546 goat anti-mouse IgG (H+L), #A11030; 1: 1, 000, Beyotime Alexa Fluor: 647 goat anti-rabbit IgG (H+L), #A0468; 647 goat anti-mouse IgG (H+L), #A0473; 488 goat anti-mouse IgG (H+L), #A0428; 1:1, 000, Oasis Biofarm: 647 goat anti-Rat IgG (H+L), #G-RT647; 647 goat anti-Guine a pig IgG, #G-GP647)] and DAPI for labeling of nuclei (1:1, 000, #4083S, CST) in 5% BSA at room temperature incubation for 1 h. The primary antibodies included rabbit anti-YAP (1:500, GTX129151, GeneTex), rabbit anti-YAP (1:100, #14074, CST), mouse anti-GFAP (1:500, MAB360, Millipore), mouse anti-NeuN (1:400, #94403S, CST), pig anti-Iba1 (1:500, OB-PGP049-01, Oasis Biofarm), rabbit anti-TDP-43 (1:100, #3448S, CST), rabbit anti-NF (1:500, ab8135, Abcam), rabbit anti-Iba1 (1:500, ab178846, Abcam), rabbit anti-CD45 (1:500, ab10558, Abcam), rabbit anti-CD206 (1:400, #24595, CST), rabbit anti-Vimentin (1:200, ET1610-39, HUABIO), rabbit anti-S100β (1:200, OB-PRB050-01, Oasis Biofarm), rabbit anti-ALDH1L1 (1:500, OB-PRB001-01, Oasis Biofarm), rabbit anti-Ki67 (1:400, #9129S, CST), rat anti-EAAT2 (1:200, OB-PRT026, Oasis Biofarm), and rabbit anti-Cleaved-caspase-3 (1:400, #9661S, CST). Images were acquired with fluorescence microscope (VS200, Olympus) or Nikon confocal microscope (Nikon A1) or Nikon single-photon and two-photon integrated confocal microscope (Nikon AX R MP), and analyzed by the ImageJ software.

### Immunohistochemistry

Brain tissue sections were fixed for 30 min and incubated in 3% hydrogen peroxide to block endogenous peroxidase activity, and then repaired with sodium citrate antigen repair solution at 90℃ for 30 min. Next, the brain tissue sections were permeabilized and blocked with 5% BSA (#V900933, Sigma-Aldrich) and 0.3% Triton X-100 (#T8200, Solarbio) at room temperature for 1.5 h. The sections were subsequently incubated overnight at 4℃ with rabbit anti-TDP-43 (1:2, 000, 10782-2-AP, Proteintech) primary antibody. The following day, after washing with PBS three times, the sections were incubated with universal secondary antibodies labeled with HRP (PV-9000, 2-step plus® Poly-HRP Anti Mouse/Rabbit IgG Detection System, ZSGB-Bio) for 1 h at room temperature. Immunoreactivity was visualized using DAB reagents (ZSGB-Bio). Finally, the nuclei were stained with hematoxylin staining solution (C0107, Beyotime), and images were captured using an Olympus VS200 microscope.

### Nissl staining

Brain tissue sections were incubated with 0.1% cresyl violet solution (#C5042-10G, Sigma) at room temperature for 10 min, followed by washing with distilled water. The sections were then dehydrated in 75%, 95% and anhydrous ethanol, cleared with xylene twice (the first time for 5 min and the second time for 10 min), and finally covered with neutral resin. Images were captured using a microscope (Olympus, VS200), and the density of Nissl bodies in the cerebral motor cortex was quantified using ImageJ software.

### Western blotting

Brain tissue or cultured cells were lysed with ice-cold RIPA solution (P0013B, Beyotime) for 30 min, and tissue lysates or cell lysates were centrifuged 12, 000 rpm for 30 min at 4℃. Then the extracted protein was mixed with the loading buffer and boiled at 100℃ for 15 min. Subsequently, the samples were separated by 8-10% SDS-PAGE gel electrophoresis and transferred to western blotting membrane (Millipore, ISEQ00010). The blotted membranes were blocked in 5% skim milk at room temperature for 1.5 h and incubated with primary antibodies overnight at 4℃. The primary antibodies included rabbit anti-YAP (1:1, 000, #14074, CST), rabbit anti-p-YAP (1:1, 000, #130084, CST), mouse anti-GFAP (1:1, 000, MAB360, Millipore), rat anti-EAAT2 (1:1, 000, OB-PRT026, Oasis Biofarm), rabbit anti-EAAT2 (1:1, 000, 22515-1-AP, Proteintech, for Figure 6I and Figure 7M), rabbit anti-β-catenin (1:1, 000, #8480S, CST), rabbit anti-β-catenin (1:100, ET1601-5, HUABIO), rabbit anti-TNF-α (1:400, GB11188-100, Servicebio), rabbit anti-Bax (1:5, 000, ET1603-34, HUABIO), rabbit anti-Bcl-2 (1:2, 000, ab182858, Abcam), rabbit anti-Bcl-2 (1:5, 000, ET1702-53, HUABIO). Rabbit anti-GAPDH (1:5, 000, ET1601-4, HUABIO) or mouse anti-β-actin (1:10, 000, EM21002, HUABIO) or rabbit anti-Lamin B1 (1: 1, 000, ab16048, Abcam) were served as loading controls. After washing in 1 x tris buffered saline with Tween 20 (TBST) for three times and incubating with appropriate secondary antibody (Goat anti-rabbit HRP, 1: 10, 000, #FDR007, Fdbio; Anti-mouse IgG HRP, 1: 10, 000, #A9044, Sigma; Goat anti-rat IgG, HRP, 1:5, 000, #RT-HRP, Oasis Biofarm) at room temperature for 1 h. The blots were then washed three times with 1 x TBST, and the protein blots were detected by Fdbio-Dura ECL kit (#FD8020, Fdbio) on the electrochemiluminescence imaging analysis system (GelViev 6000Plus, BLT). The gray values were subsequently analyzed using ImageJ software.

### Cytosol-nuclei fractionation

We used the Minte™ Cytosolic and Nuclear Extraction kit for Frozen/Fresh Tissues (#NT-032, Invent Biotechnologies) to dissociate cytoplasmic and nuclear proteins from the cortex according to manufacturer's instructions. The fractions were analyzed by SDS-PAGE and Western blotting with specific antibodies.

### RNA extraction and quantitative real-time PCR (qPCR)

Total RNA was extracted from the cerebral cortex, Neuro-2a (N2a) cells, primary cultured astrocytes and neurons using RNA-easy Isolation Reagent (R701-01, Vazyme) according to the manufacturer's instructions. The expression levels of *EAAT2*,* β-catenin*,* TNF-α*,* IL-1β*,* IL-6*,* CXCL10*,* CD206*,* Wnt1*,* Wnt2*,* Wnt3a*,* Wnt4*,* Wnt5a and Wnt7a* mRNA were quantified using ChamQ Universal SYBR qPCR Master Mix (Q711-02, Vazyme) on a CFX96 Touch Real-Time PCR Detection System (Bio-Rad). *β-actin* was used as endogenous control. Δ CT = CT gene - CT reference was used to refer to the relative mRNA expression, and 2^-ΔΔ CT^ method was used to calculate the change of gene expression multiple. The primers used in this study were synthesized by Tsingke Biotechnology Company and expressed as followed:

*EAAT2* primer: forward, 5'-CTGATGTGGTCATGTTGATAGCC-3', reverse, 5'-AACTGGAGATGATAAGAGGGAGG-3'.

*β-catenin* primer: forward, 5'-ATGGAGCCGGACAGAAAAGC-3', reverse, 5'-TGGGAGGTGTCAACATCTTCTT-3'.

*TNF-α* primer: forward, 5'-TCTCATGCACCACCATCAAGGACT-3', reverse, 5'- ACCACTCTCCCTTTGCAGAACTCA -3'.

*IL-1β* primer: forward, 5'-AAGGGCTGCTTCCAAACCTTTGAC-3', reverse, 5'-ATACTGCCTGCCTGAAGCTCTTGT-3'.

*IL-6* primer: forward, 5'-ATCCAGTTGCCTTCTTGGGACTGA-3', reverse, 5'-TAAGCCTCCGACTTGTGAAGTGGT-3'.

*CXCL10* primer: forward, 5'-GGAAATCGTGCGTGACATTA-3', reverse, 5'-AGGAAGGAAGGCTGGAAGAG-3'.

*CD206* primer: forward, 5'-TCAGCTATTGGACGCGAGGCA-3', reverse, 5'-TCCGGGTTGCAAGTTGCCGT-3'.

*Wnt1* primer: forward, 5'-GGTTTCTACTACGTTGCTACTGG-3', reverse, 5'- GGAATCCGTCAACAGGTTCGT-3'.

*Wnt2* primer: forward, 5'-CTCGGTGGAATCTGGCTCTG-3', reverse, 5'- CACATTGTCACACATCACCCT-3'.

*Wnt3a* primer: forward, 5'-CAGGAACTACGTGGAGATCATGC-3', reverse, 5'- CGTGTCACTGCGAAAGCTACT-3'.

*Wnt4* primer: forward, 5'-GTCAGGATGCTCGGACAACAT-3', reverse, 5'- CACGTCTTTACCTCGCAGGA-3'.

*Wnt5a* primer: forward, 5'-CTGCGGAGACAACATCGACTA-3', reverse, 5'- CGTGGATTCGTTCCCTTTCTCTA-3'.

*Wnt7a* primer: forward, 5'-TGAACTTACACAATAACGAGGCG-3', reverse, 5'- GTGGTCCAGCACGTCTTAGT-3'.

*β-actin* primer: forward, 5'-GGCACCACACCTTCTACAATG-3', reverse, 5'-GGGGTGTTGAAGGTCTCAAAC-3'.

### Cell lines and transfection

Mouse Neuro-2a (N2a) cells were obtained from Procell (CL-0168) and cultured in DMEM (C11995500BT, Gibco), containing 10% fetal bovine serum (FBS, S-FBS-MX-015, SERANA) and 1% penicillin/streptomycin (PS, C0222, Beyotime). GFP-GA_5_ or GFP-GA_100_ plasmids (kindly provided by Prof. Chengyong Shen, Zhejiang University) were separately transfected into N2a cells using Lipofectamine^®^ 3000 Transfection Kit (L3000-015, Invitrogen) according to the manufacturer's protocol. Cells and conditioned medium (CM) were collected after transfection for subsequent experiments.

### Primary neuron culture

For primary neuron culture, we isolated cells from the cortex and hippocampus of wild- type (WT) mouse embryos at days 14-16 of gestation. Cells were digested with 0.25% trypsin (C0201-500 ml, Beyotime) for 12 min at 37°C, resuspended with DMEM containing 10% FBS and 1% PS, and plated on poly-L-lysine coated six-well plates or coverslips. After 2-4 h, the medium was replaced with neuronal basal medium [Neurobasal medium (21103049, Gibco)/2% B27 (17504044, Gibco)/1% GlutaMAX (35050-061, Gibco)/1% PS]. Neurons were treated with 2 µM cytosine arabinoside at 2 days *in vitro* (DIV2) for 24 h to suppress glial proliferation. The medium was changed once every 2 days. To mimic the pathology of ALS *in vitro*, neurons at DIV3 were transduced with AAV-GFP-GA_5_ virus or AAV-GFP-GA_100_ virus at multiplicities of infection (MOIs) of 2×10⁵ or 4×10⁵ vg/cell, and the GFP expression was confirmed 48 h post-transduction 53. CM from the transduced neurons was collected every 2 days.

### Cell viability assay

Cell viability was assayed by Cell Counting Kit-8 (CCK-8, FD3788, Fdbio). Briefly, primary cultured neurons were inoculated into 96-well plates coated with poly-L-lysine. The cell density in each well was approximately 10, 000-50, 000 cells. Primary cultured neurons at DIV3 were transduced with AAV-GFP-GA_100_ virus. 48 h post-transduction, GFP expression was confirmed, and neurons were treated with CM from YAP^+/+^ or YAP^-/-^ astrocytes for 24 h. Subsequently, 10 µl of CCK-8 solution was added to each well and incubated at 37°C for 2 h. The absorbance of each well was assessed at 450 nm using a microplate reader (Multiskan FC, Thermo Scientific).

### Astrocyte culture

YAP^+/+^ and YAP^-/-^ astrocyte cultures were derived from the cortex and hippocampus of YAP^f/f^ and YAP^GFAP^-CKO mice, respectively, as described previously 54. Briefly, the cortex and hippocampus of P1-P3 mice were minced, digested with 0.25% trypsin (C0201-500 ml, Beyotime) for 12 min at 37℃, and mechanically disrupted to separate into single-cell suspensions. Cells were then cultured in DMEM containing 10% FBS and 1% PS in a humidified atmosphere with 5% CO_2_ at 37℃. After 6-10 days of culture, microglia and oligodendrocytes were removed by shaking at 250 rpm for 4-6 h. Then, the astrocytes were plated on poly-L-lysine coated dishes or coverslips for subsequent experiments.

### Collecting astrocyte conditioned medium (ACM)

Astrocyte cultures were prepared from YAP^f/f^ and YAP^GFAP^-CKO mice. Cultures were maintained at 37°C and 5% CO_2_ in DMEM containing 10% FBS and 1% PS. Cultures were passaged to reach confluence and contained >95% glial fibrillary acidic protein (GFAP)-positive astrocytes. ACM was collected every 2 days, filtered and stored at -80℃ for treatment of N2a cells. For ACM used in primary cultures for neuronal survival experiments, neuronal basal medium was substituted for the previous medium and ACM was collected every 2 days, filtered, and stored at -80°C 55.

### Flow cytometry analysis

The apoptosis rate of transfected N2a cells was analyzed by the Annexin V-APC/PI apoptosis kit (AP107-30-kit, Multi Sciences). GFP-GA_5_ or GFP-GA_100_ plasmids were transfected into N2a cells using the Lipofectamine^®^ 3000 Transfection Kit (L3000-015, Invitrogen), and then conditioned medium from YAP^+/+^ or YAP^-/-^ astrocytes was used to culture the transfected N2a cells. Cells were collected and washed, resuspended with 500 µl 1x binding buffer, and then incubated with Annexin V-APC and PI staining solution. After incubation in the dark for 5 min at room temperature, the apoptosis rate of cells was detected by flow cytometer (Beckman, CytoFLEX S) and analyzed using FlowJo software.

### Glutamate uptake assay

The glutamate clearance capacity was evaluated, and the glutamate uptake assay was determined according to the manufacturer's instructions (#BC1580, Solarbio) and the previous description 54. In brief, the medium for YAP^+/+^ and YAP^-/-^ astrocytes was replaced with L-glutamine-free DMEM (Gibco, 10313-021) containing 2 mM glutamate (H-Glu-OH, GA10750, GlpBio). After 30 min of culture, 100 µl of astrocyte culture supernatant was collected and mixed with the reagents provided by the kit. Then, 100 µl of mixed solution was transferred to each well of a 96-well culture plate. The absorbance of the solution was measured at 340 nm using an enzyme-labeled instrument (Infinite M Plex, Tecan). A standard curve of glutamate concentration was constructed using culture medium with known glutamic acid concentration and the glutamate content was calculated according to the manufacturer's instructions.

### Injection of drugs and reagents

After the model of C9orf72-poly-GA was established, LDN-212320 (HY-12741, MedChemExpress) was dissolved in 0.9% NaCl containing 1% DMSO, 1% polyethylene glycol 400, and 0.2% Tween 80 and administered continuously for two weeks starting at P75 (40 mg/kg intraperitoneally). XMU-MP-1 (GC11245, GlpBio or GY-100526, MedChemExpress) was dissolved in DMSO and continuously administered for two weeks starting at P75 (1 mg/kg intraperitoneally). Behavioral analysis was subsequently performed.

MSAB (HY-120697, MedChemExpress) was dissolved in DMSO and applied at a concentration of 10 µM for 20 h, unless otherwise specified.

### Data analysis and statistics

All data were presented as mean ± SEM from at least three independent experiments. Statistical analysis was performed using GraphPad Prism 8.0.1 and ImageJ. Comparisons between two groups were performed using an unpaired two-tailed Student's *t*-test; for comparisons between multiple groups, one-way ANOVA or two-way ANOVA with Tukey's multiple comparisons test was used.* P<0.05* was considered statistically significant and details of each statistical test were described in the Figure legends.

## Results

### YAP is upregulated and activated in astrocytes, but not in neurons or microglia, and YAP knockout in astrocytes exacerbates motor deficits in C9orf72-poly-GA mice

To explore the potential role of YAP in ALS, we established the C9orf72-poly-GA pathological ALS mouse model as described previously (ALS mice) 51. AAV-GFP-GA_5_ and AAV-GFP-GA_100_ viruses were injected into the cerebral ventricles of P0 neonatal pups, respectively (Supplementary Figure S1A-B). To determine whether AAV-GFP-GA_100_-infected mice exhibited ALS-associated phenotypes, behavioral analyses were performed at 3 months of age, followed by histological and biochemical analyses at 104 days (Supplementary Figure S1B). As expected, in the tail-suspension test, ALS mice injected with AAV-GFP-GA_100_ virus exhibited hind limb clasping and persistent trembling, while control mice injected with AAV-GFP-GA_5_ virus did not display these phenotypes (Supplementary Figure S1C-D). Subsequently, in the balance beam test, ALS mice exhibited significantly increased hind limb slips compared to control mice, indicating serious deficits in motor balance in mice expressing poly-GA_100_ (Supplementary Figure S1E). On day 104, it was found that the cerebral cortex of mice injected with AAV-GFP-GA_5_ and AAV-GFP-GA_100_ showed abundant GFP fluorescence signals (Supplementary Figure S1F). Compared to control mice, the brains of ALS mice showed abundant GFP-GA_100_ puncta (arrow) (Supplementary Figure S1F), similar to the neuronal cytoplasmic inclusion bodies observed in the brains of C9orf72-ALS/FTD patients 51. In contrast, these puncta were scarcely observed in the brains of control mice. In summary, these results suggest that an ALS mouse model of C9orf72-poly-GA has been successfully established.

**Figure 1:**
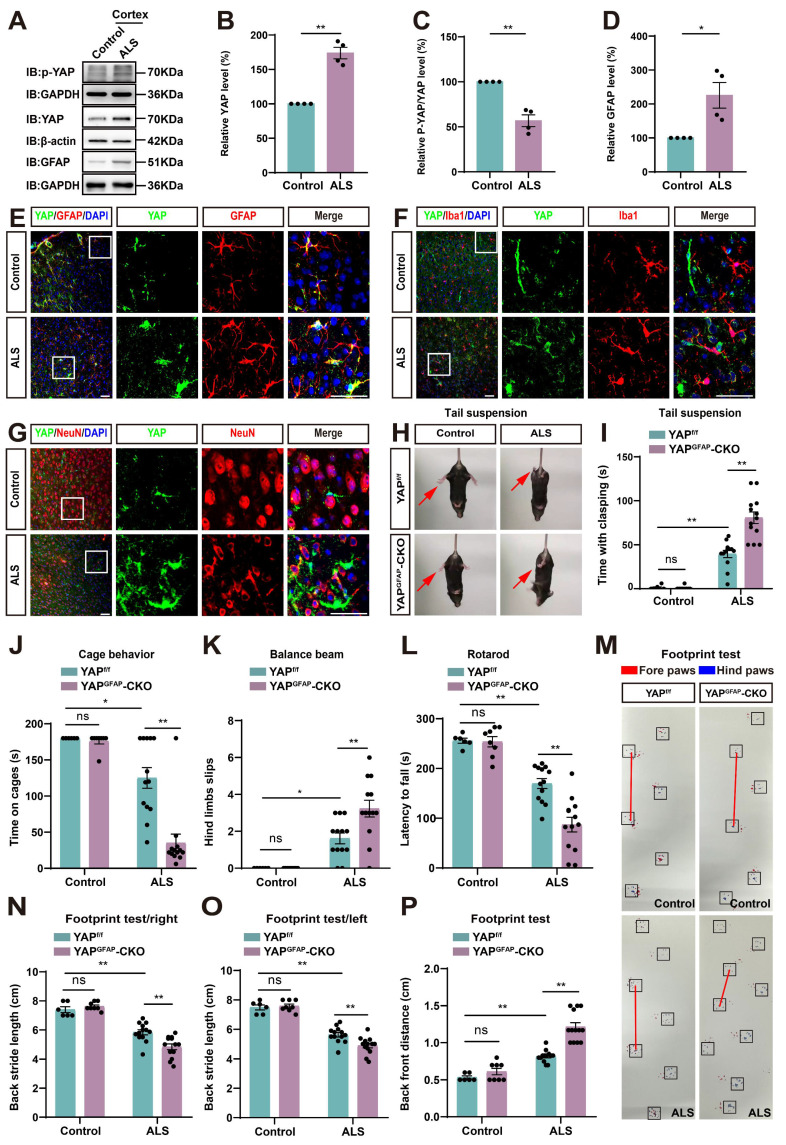
** YAP is upregulated and activated in astrocytes, but not in neurons or microglia, and YAP knockout in astrocytes exacerbates motor deficits in C9orf72-poly-GA mice.** (**A**) Western blot analysis of YAP, p-YAP/YAP and GFAP expression in the cortex of control mice and C9orf72-poly-GA mice (ALS mice). (**B-D**) Quantitative analysis of the relative YAP (**B**), p-YAP/YAP (**C**) and GFAP (**D**) expression levels as shown in (**A**) (n = 4, normalized to control mice, Student's *t*-test). (**E-G**) Representative staining images of YAP (green) and GFAP (red) (**E**), YAP (green) and Iba1 (red) (**F**), and YAP (green) and NeuN (red) (**G**) in the motor cortex of control mice and ALS mice. (**H**) Representative images of YAP^f/f^ and YAP^GFAP^-CKO control mice, YAP^f/f^ ALS and YAP^GFAP^-CKO ALS mice in tail-suspension test. The red arrows indicate the hind limbs of mice. (**I**) Quantitative analysis of hind limb clasping time of YAP^f/f^ and YAP^GFAP^-CKO control mice, YAP^f/f^ ALS and YAP^GFAP^-CKO ALS mice within 3 min in tail-suspension test. (**J**) Quantitative analysis of time keeping on the edges of cages of YAP^f/f^ and YAP^GFAP^-CKO control mice, YAP^f/f^ ALS and YAP^GFAP^-CKO ALS mice. (**K**) Quantitative analysis of the numbers of hind limb foot slips in the balance beam test in YAP^f/f^ and YAP^GFAP^-CKO control mice, YAP^f/f^ ALS and YAP^GFAP^-CKO ALS mice. (**L**) Quantitative analysis of the latency to fall from the accelerated rotating rod of YAP^f/f^ and YAP^GFAP^-CKO control mice, YAP^f/f^ ALS and YAP^GFAP^-CKO ALS mice. (**M**) Representative images of YAP^f/f^ and YAP^GFAP^-CKO control mice, YAP^f/f^ ALS and YAP^GFAP^-CKO ALS mice in footprint test. Fore paws (red), hind paws (blue). Black squares indicate localization of hind paws. The red line indicates the distance between the hind paws. (**N-P**) Quantitative analysis of back stride length (right) (**N**), back stride length (left) (**O**), and back front distance (**P**) of YAP^f/f^ and YAP^GFAP^-CKO control mice, YAP^f/f^ ALS and YAP^GFAP^-CKO ALS mice in footprint test. For behavioral tests, YAP^f/f^ control mice, n = 6 each group; YAP^GFAP^-CKO control mice, n = 8 each group; YAP^f/f^ ALS and YAP^GFAP^-CKO ALS mice, n = 13 each group. Scale bars, 50 µm. Data were presented as mean ± SEM. Two-way ANOVA with Tukey's multiple comparisons test unless otherwise indicated, n.s., not significant (*p > 0.05*),^ *^p < 0.05, ^**^p < 0.01.

Next, we examined the expression pattern of YAP in ALS mice. Cortex tissues were collected from control and ALS mice. Western blot analysis showed that YAP expression was significantly increased in the cortex of ALS mice (Figure 1A-B), while the relative ratio of phosphorylated YAP (p-YAP)/YAP was significantly decreased (Figure 1A, C). Additionally, the expression of GFAP (astrocyte marker) was also significantly upregulated in the cortex of ALS mice (Figure 1A, D). These results indicate that YAP is upregulated and activated in the cortex of C9orf72-poly-GA mice. Furthermore, double immunostaining of YAP with several cellular markers, such as GFAP (astrocyte marker), Iba1 (microglia marker) and NeuN (neuronal marker) in the motor cortex of control mice and ALS mice showed that YAP was predominantly expressed in GFAP^+^ astrocytes (Figure 1E), but not in Iba1^+^ microglia (Figure 1F) or NeuN^+^ neurons (Figure 1G), and YAP was primarily located in the nucleus of GFAP^+^ astrocytes in ALS mice (Figure 1E), indicating activation of YAP signaling in astrocytes. Taken together, these results suggest that YAP is upregulated and activated in motor cortex astrocytes of C9orf72-poly-GA mice.

To further study the role of astrocytic YAP in ALS, YAP^GFAP^-CKO mice with conditional knockout of YAP in astrocytes were generated (Supplementary Figure S2A). In YAP^GFAP^-CKO mice, YAP expression was significantly decreased in brain regions, including the cortex, hippocampus, cerebellum, and spinal cord (Supplementary Figure S2B-C). Immunostaining also showed that YAP signal was not detected in GFAP^+^ astrocytes in the cerebral cortex of YAP^GFAP^-CKO mice and in primary cultured astrocytes (Supplementary Figure S2D-E). However, no significant difference in body size was observed between YAP^f/f^ and YAP^GFAP^-CKO mice (Supplementary Figure S2F). Behavioral tests, including the tail-suspension test (Supplementary Figure S2G-H), cage behavior test (Supplementary Figure S2I), balance beam test (Supplementary Figure S2J), and rotarod test (Supplementary Figure S2K), showed that YAP deficiency in astrocytes had no significant effect on motor functions of mice. These results suggest that the knockout of astrocytic YAP does not affect the motor function of the mice.

Next, we examined the potential phenotypic differences between YAP^f/f^ C9orf72-poly-GA mice (YAP^f/f^ ALS mice) and YAP^GFAP^-CKO C9orf72-poly-GA mice (YAP^GFAP^-CKO ALS mice). The tail-suspension test showed that compared to YAP^f/f^ ALS mice, YAP^GFAP^-CKO ALS mice exhibited more pronounced hind limb clasping and continuous trembling (Figure 1H-I). Furthermore, the cage behavior test showed that YAP^GFAP^-CKO ALS mice were more likely to fall within 3 min while walking along the edge of the cage (Figure 1J), indicating more severe deficits in motor balance in YAP^GFAP^-CKO ALS mice. To further confirm these results, we used three widely utilized assays to test motor function: the balance beam test, rotarod test, and footprint test. The results showed that the hind limb slips on the balance beam were significantly increased in YAP^GFAP^-CKO ALS mice (Figure 1K), and the latency to fall off the rotating rod was significantly decreased in YAP^GFAP^-CKO ALS mice (Figure 1L), and the footprint test showed more pronounced gait abnormalities in YAP^GFAP^-CKO ALS mice (Figure 1M-P). Taken together, these results suggest that YAP knockout in astrocytes exacerbates motor deficits in C9orf72-poly-GA mice.

### YAP knockout in astrocytes exacerbates neurodegeneration and pathological TDP-43 cytoplasmic translocation in C9orf72-poly-GA mice

**Figure 2:**
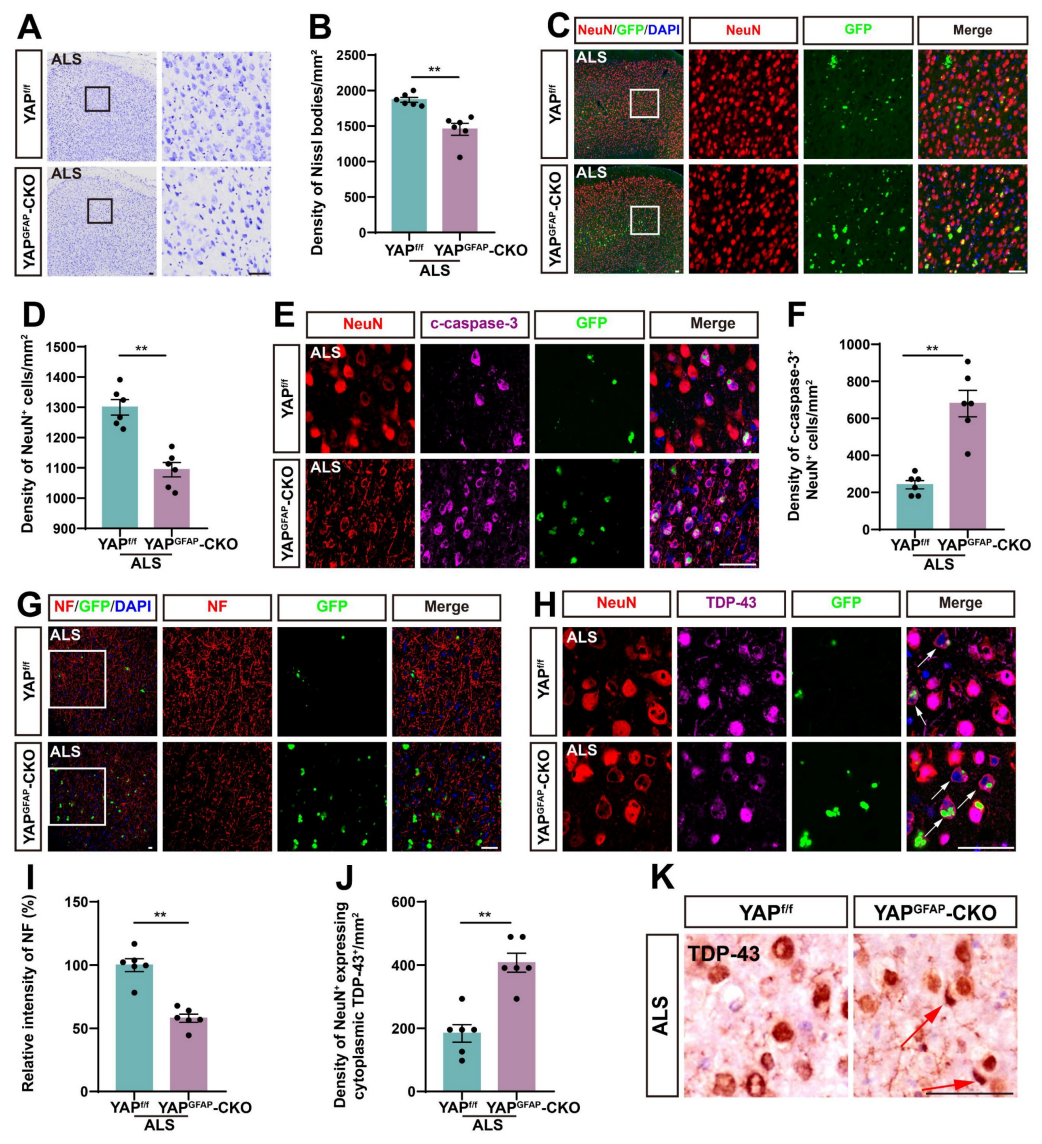
** YAP knockout in astrocytes exacerbates neurodegeneration and pathological TDP-43 cytoplasmic translocation in C9orf72-poly-GA mice.** (**A**, **C**) Representative staining images of Nissl staining (**A**) and NeuN (red) (**C**) in the motor cortex of YAP^f/f^ ALS mice and YAP^GFAP^-CKO ALS mice. (**B, D**) Quantitative analysis of the density of Nissl bodies (**B**) and NeuN^+^ cells (**D**) as shown in (**A**) and (**C**), respectively (n = 6 each group). (**E**) Representative staining images of NeuN (red) and c-caspase-3 (far-red) in the motor cortex of YAP^f/f^ ALS mice and YAP^GFAP^-CKO ALS mice. (**F**) Quantitative analysis of the density of c-caspase-3^+^ in NeuN^+^ neurons as shown in (**E**) (n = 6 each group). (**G-H**) Representative staining images of NF (red) (**G**) and TDP-43 (far-red) (**H**) in the motor cortex of YAP^f/f^ ALS mice and YAP^GFAP^-CKO ALS mice. White arrows indicate cytoplasmic TDP-43 expression. (**I**) Quantitative analysis of the relative intensity of NF as shown in (**G**) (n = 6 each group). (**J**) Quantitative analysis of the density of cytoplasmic TDP-43 in NeuN^+^ neurons as shown in (**H**) (n = 6 each group). (**K**) Immunohistochemistry of TDP-43 aggregation in the motor cortex of YAP^f/f^ ALS mice and YAP^GFAP^-CKO ALS mice. Red arrows indicate the cytoplasmic inclusion bodies of TDP-43. Scale bars, 50 µm. Data were presented as mean ± SEM. Student's *t*-test,^ **^p < 0.01.

Since motor behavioral deficits in ALS result from the neurodegeneration of motor neurons, we next examined the loss of neurons in the motor cortex of YAP^GFAP^-CKO ALS mice. Notably, both Nissl staining and immunostaining of NeuN showed a significant reduction in the density of Nissl bodies (Figure 2A-B) and NeuN^+^ neurons (Figure 2C-D) in the motor cortex of YAP^GFAP^-CKO ALS mice, compared to YAP^f/f^ ALS mice. In addition, double immunostaining for NeuN and Cleaved-caspase-3 (c-caspase-3) further demonstrated a significant increase in neuronal apoptosis in the motor cortex of YAP^GFAP^-CKO ALS mice (Figure 2E-F).

Previous studies have shown that neurofilaments, as the main intermediate filament system in mature neurons, are essential for neuronal function, and abnormalities in neurofilament can directly lead to motor neuron dysfunction 56-58. Immunostaining for NF (neurofilament heavy polypeptide) showed that the intensity of NF was significantly lower in YAP^GFAP^-CKO ALS mice (Figure 2G, I). Collectively, these results suggest that knockout of astrocytic YAP exacerbates neurodegeneration in C9orf72-poly-GA mice. TDP-43 cytoplasmic inclusions are major neuropathological feature of C9orf72-ALS/FTD 51, 59-61. Therefore, we examined TDP-43 translocation in the motor cortex of C9orf72-poly-GA mice. As expected, double immunostaining showed a significant increase in cytoplasmic TDP-43 aggregation in the neurons of YAP^GFAP^-CKO ALS mice (Figure 2H, J-K). Taken together, these results suggest that YAP knockout in astrocytes exacerbates neurodegeneration and pathological TDP-43 cytoplasmic translocation in C9orf72-poly-GA mice.

### YAP knockout in astrocytes exacerbates the inflammatory infiltration in the motor cortex of C9orf72-poly-GA mice

**Figure 3:**
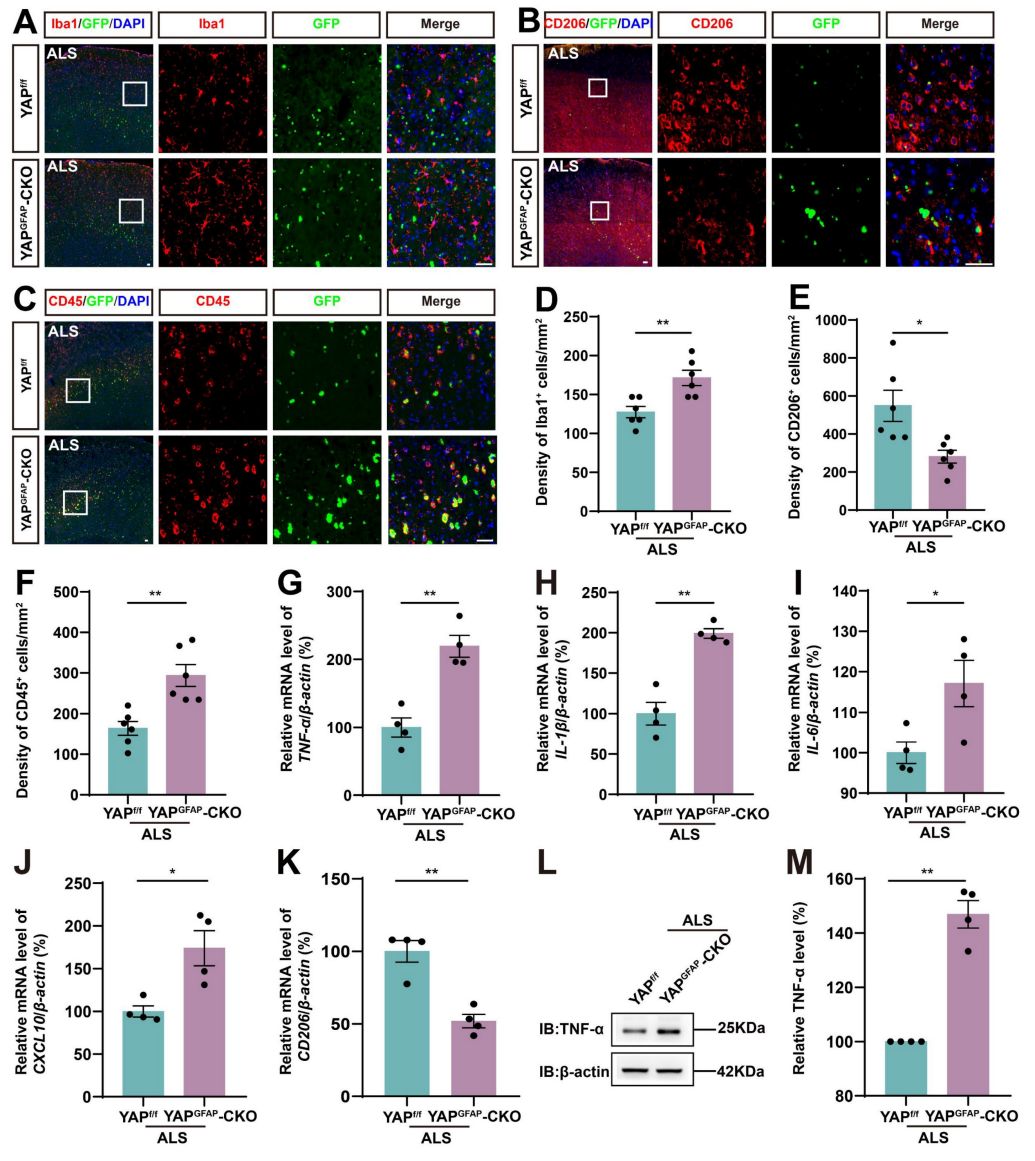
** YAP knockout in astrocytes exacerbates the inflammatory infiltration in the motor cortex of C9orf72-poly-GA mice.** (**A**-**C**) Representative staining images of Iba1(red) (**A**), CD206 (red) (**B**)and CD45 (red) (**C**) in the motor cortex of YAP^f/f^ ALS mice and YAP^GFAP^-CKO ALS mice. (**D-F**) Quantitative analysis of the density of Iba1^+^ cells (**D**), CD206^+^ cells (**E**) and CD45^+^ cells (**F**) as shown in (**A**), (**B**) and (**C**), respectively (n = 6 each group). (**G-K**) qPCR analysis of the relative mRNA levels of *TNF-α* (**G**), *IL-1β* (**H**), *IL-6* (**I**), *CXCL10* (**J**) and *CD206* (**K**) in the cortex of YAP^f/f^ ALS mice and YAP^GFAP^-CKO ALS mice (n = 4 each group). (**L**) Western blot analysis of TNF-α expression in the cortex of YAP^f/f^ ALS mice and YAP^GFAP^-CKO ALS mice. (**M**) Quantitative analysis of the relative TNF-α expression level as shown in (**L**) (n = 4 each group, normalized to YAP^f/f^ ALS mice). Scale bars, 50 µm. Data were presented as mean ± SEM. Student's *t*-test,^*^p < 0.05,^**^p < 0.01.

Neuroinflammation is a prominent pathological manifestation of ALS, characterized by microglia activation, astrocyte proliferation, and monocyte and T-cell infiltration 62. Therefore, we next examined the inflammatory infiltration in YAP^GFAP^-CKO ALS mice. The results showed that Iba1^+^ microglia were activated, and the density of Iba1^+^ microglia was significantly increased in the motor cortex of YAP^GFAP^-CKO ALS mice (Figure 3A, D). M2 macrophages produce high levels of anti-inflammatory cytokines and neurotrophic factors 63. We found the density of CD206^+^ (an M2 macrophage marker) cells was significantly decreased in YAP^GFAP^-CKO ALS mice (Figure 3B, E). Similarly, immunostaining of CD45^+^ (a marker of common leukocyte antigen) showed that a significant increase in the density of CD45^+^ cells in the motor cortex of YAP^GFAP^-CKO ALS mice (Figure 3C, F). Additionally, we examined the expression of inflammatory factors in the cortex of these mice. The qPCR results showed that pro-inflammatory markers, including *TNF-α* (Figure 3G), *IL-1β* (Figure 3H), *IL-6* (Figure 3I), and *CXCL10* (Figure 3J) mRNA, were significantly upregulated in the cortex of YAP^GFAP^-CKO ALS mice, whereas the mRNA of anti-inflammatory marker *CD206* (Figure 3K) was significantly downregulated. Western blot analysis further confirmed that the expression of TNF-α was upregulated in the cortex of YAP^GFAP^-CKO ALS mice (Figure 3L-M). Taken together, these results suggest that the inflammatory infiltration is exacerbated in the motor cortex of YAP^GFAP^-CKO ALS mice.

### The density and proliferation of astrocytes are reduced in the motor cortex of YAP^GFAP^-CKO ALS mice

**Figure 4:**
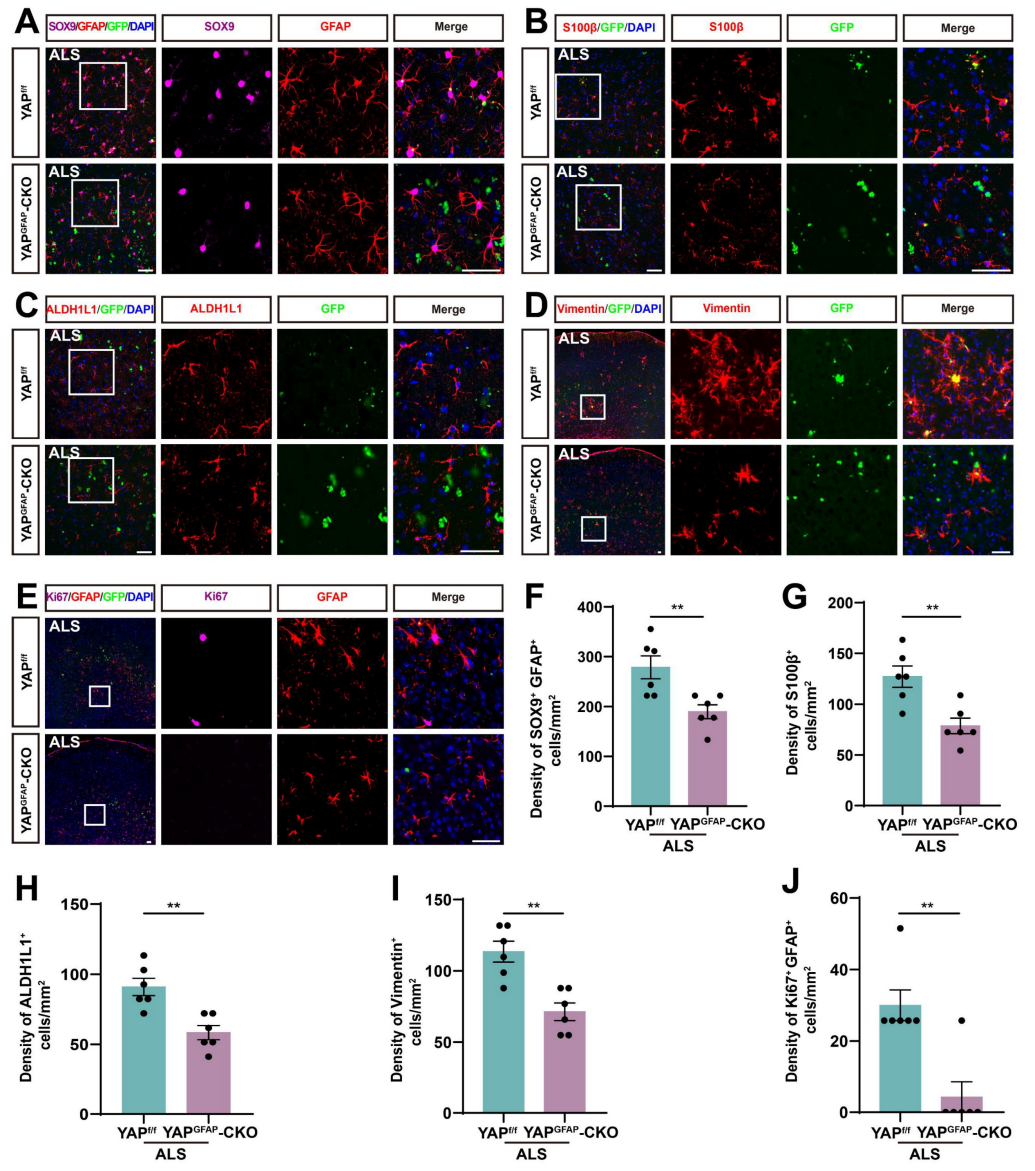
** The density and proliferation of astrocytes are reduced in the motor cortex of YAP^GFAP^-CKO ALS mice.** (**A**) Representative staining images of SOX9 (far-red) and GFAP (red) in the motor cortex of YAP^f/f^ ALS mice and YAP^GFAP^-CKO ALS mice. (**B-D**) Representative staining images of S100β (red) (**B**), ALDH1L1 (red) (**C**)and Vimentin (red) (**D**) in the motor cortex of YAP^f/f^ ALS mice and YAP^GFAP^-CKO ALS mice. (**E**) Representative staining images of Ki67 (far-red) and GFAP (red) in the motor cortex of YAP^f/f^ ALS mice and YAP^GFAP^-CKO ALS mice. (**F**) Quantitative analysis of the density of SOX9^ +^ and GFAP^+^ double positive cells as shown in (**A**) (n = 6 each group). (**G-I**) Quantitative analysis of the density of S100β^+^ cells (**G**), ALDH1L1^+^ cells (**H**) and Vimentin^+^ cells (**I**) as shown in (**B**), (**C**) and (**D**), respectively (n = 6 each group). (**J**) Quantitative analysis of Ki67^+^ and GFAP^+^ cells as shown in (**E**) (n = 6 each group). Scale bars, 50 µm. Data were presented as mean ± SEM. Student's *t*-test, ^**^p < 0.01.

Studies have shown that reactive astrocytes are a key feature of ALS 64, 65. To investigate astrocyte changes in the motor cortex of YAP^GFAP^-CKO ALS mice, we performed immunostaining for astrocyte markers. SOX9, a transcription factor known to support the development of glial cells in the peripheral nervous system, has been reported to promote differentiation into astrocytes 66, 67. Therefore, we performed double immunostaining for SOX9 and GFAP in the motor cortex of YAP^f/f^ ALS mice and YAP^GFAP^-CKO ALS mice. The results showed that the density of SOX9^+^ astrocytes was significantly reduced in the motor cortex of YAP^GFAP^-CKO ALS mice (Figure 4A, F). To further confirm these results, we stained for other astrocyte markers, including S100β, ALDH1L1 and Vimentin, and found that the density of S100β^+^ (Figure 4B, G), ALDH1L1^+^ (Figure 4C, H) and Vimentin^+^ (Figure 4D, I) astrocytes was significantly decreased in the motor cortex of YAP^GFAP^-CKO ALS mice, suggesting that the density of astrocytes was reduced in the motor cortex of YAP^GFAP^-CKO ALS mice. In addition, double immunostaining for Ki67 (a cell proliferation marker) and GFAP further showed that astrocytic proliferation was significantly reduced in the motor cortex of YAP^GFAP^-CKO ALS mice (Figure 4E, J). Taken together, these results suggest that YAP knockout in astrocytes reduces the density and proliferation of astrocytes in the motor cortex of YAP^GFAP^-CKO ALS mice.

### Astrocytic EAAT2 expression is downregulated in the motor cortex of YAP^GFAP^-CKO ALS mice through downregulating β-catenin signaling

**Figure 5:**
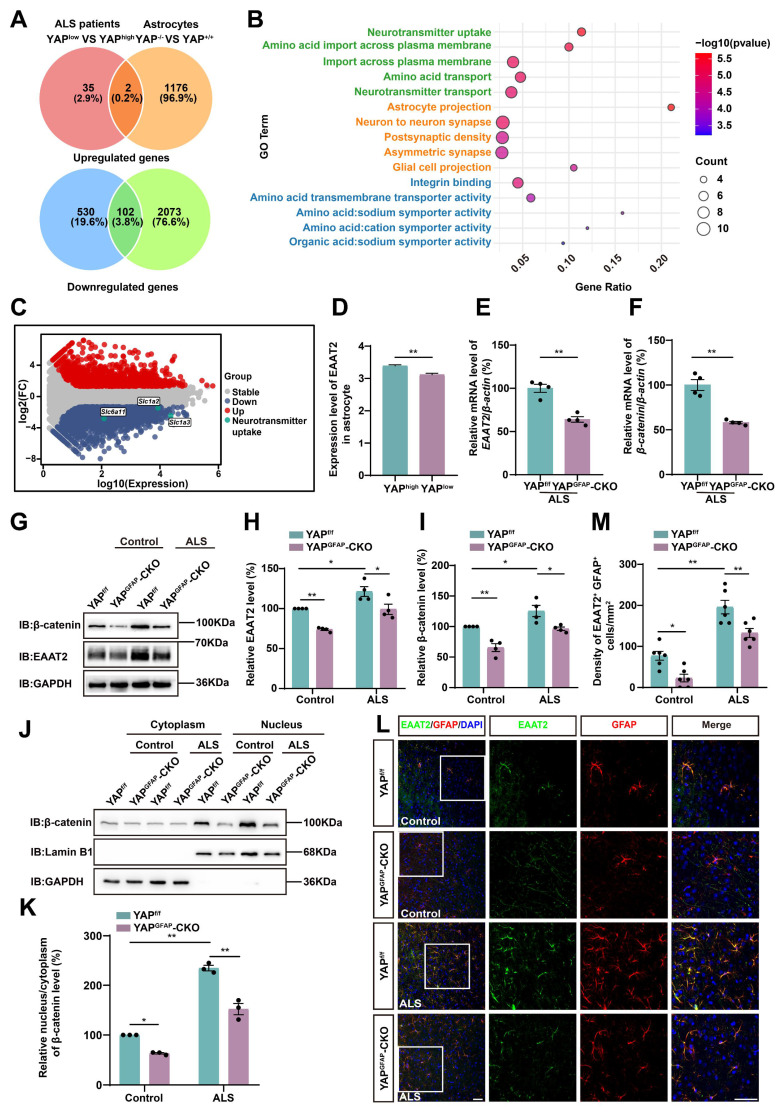
** Astrocytic EAAT2 expression is downregulated in the motor cortex of YAP^GFAP^-CKO ALS mice through downregulating β-catenin signaling.** (**A**) Overlapping differentially expressed genes between the C9orf72-ALS/FTD patient (snRNA-seq) database and RNA-seq from YAP^-/-^ and YAP^+/+^ astrocytes, including upregulated and downregulated genes. (**B**) Gene ontology (GO) enrichment of overlapping downregulated differentially expressed genes between C9orf72-ALS/FTD patient (snRNA-seq) database and RNA-seq results from YAP^+/+^ and YAP^-/-^astrocytes. (**C**) Differential gene MA-plot of YAP^+/+^ and YAP^-/-^ astrocytes. (**D**) Expression of EAAT2 in the motor cortex of C9orf72-ALS/FTD patients with high YAP expression and low YAP expression (high YAP expression group, n = 809; low YAP expression group, n = 776; Student's *t*-test). (**E**-**F**) qPCR analysis of the relative mRNA levels of *EAAT2* and *β-catenin* in the cortex of YAP^f/f^ ALS mice and YAP^GFAP^-CKO ALS mice (n = 4 each group, Student's *t*-test). (**G**) Western blot analysis of EAAT2 and β-catenin expression in the cortex of YAP^f/f^ and YAP^GFAP^-CKO control mice, YAP^f/f^ ALS and YAP^GFAP^-CKO ALS mice. (**H-I**) Quantitative analysis of the relative EAAT2 (**H**) and β-catenin (**I**) expression levels as shown in (**G**) (n = 4 each group, normalized to YAP^f/f^ control mice). (**J**) Western blot analysis of the cytoplasmic and nuclear expression of β-catenin in the cortex of YAP^f/f^ and YAP^GFAP^-CKO control mice, YAP^f/f^ ALS and YAP^GFAP^-CKO ALS mice. (**K**) Quantitative analysis of the relative nucleus/cytoplasm ratio of β-catenin expression level as shown in (**J**) (n = 3 each group, normalized to YAP^f/f^ control mice). (**L**) Representative staining images of EAAT2 (green) and GFAP (red) in the motor cortex of YAP^f/f^ and YAP^GFAP^-CKO control mice, YAP^f/f^ ALS and YAP^GFAP^-CKO ALS mice. (**M**) Quantitative analysis of the density of EAAT2^+^ and GFAP^+^ double positive cells as shown in (**L**) (n = 6 each group). Scale bars, 50 µm. Data were presented as mean ± SEM. Two-way ANOVA with Tukey's multiple comparisons test unless otherwise indicated,^ *^p < 0.05,^**^p < 0.01.

To investigate the downstream effector mechanisms through which astrocytic YAP regulates neuronal loss and motor deficits in C9orf72-poly-GA mice, we analyzed single-nucleus transcriptomic (snRNA-seq) data from motor cortex samples of C9orf72-ALS/FTD patients with low and high YAP expression 68, along with RNA sequencing (RNA-seq) results from cultured YAP^+/+^ and YAP^-/-^ (YAP knockout) astrocytes 54. We then identified their overlapping differentially expressed genes, including both upregulated and downregulated genes (Figure 5A). The results showed that there were 2 upregulated genes and 102 downregulated genes. Since YAP is believed to be a transcriptional activator, it is unlikely to directly suppress gene expression. Therefore, we next performed Gene Ontology (GO) enrichment analysis on these 102 downregulated genes and found that the most significantly altered pathway was related to neurotransmitter uptake (Figure 5B). The M-versus-A plot (MA-plot) for YAP^+/+^ and YAP^-/-^ astrocytes showed that genes related to neurotransmitter uptake, such as *Slc6a11*, *Slc1a2* (*EAAT2*) and *Slc1a3* (*EAAT1*), were downregulated (Figure 5C). Given that the loss of EAAT2 protein and function significantly contributes to excitotoxicity in ALS 8, and that EAAT2 is responsible for clearing approximately 90% of glutamate from the synaptic cleft and plays a key role in regulating excitotoxicity and neuronal death 69, 70, we further examined EAAT2 expression in the motor cortex of C9orf72-ALS/FTD patients. The results showed that EAAT2 expression was significantly downregulated in the cortex of patients with low YAP expression compared to those with high YAP expression (Figure 5D). Recent studies have shown that YAP signaling in astrocytes promotes EAAT2 expression by regulating the transcriptional activity of β-catenin 54. To further explore whether the deletion of astrocytic YAP in the motor cortex of C9orf72-poly-GA mice also reduced the expression of EAAT2 through decreased transcriptional activity of β-catenin, we conducted qPCR analysis. The results confirmed a reduction in the levels of *EAAT2* and *β-catenin* mRNA in YAP^GFAP^-CKO ALS mice (Figure 5E-F). Western blot analysis further showed that EAAT2 and β-catenin protein levels were significantly downregulated in YAP^GFAP^-CKO ALS mice (Figure 5G-I). In addition, cytosol-nuclei fractionation experiments showed that the nucleus/cytoplasm ratio of β-catenin was significantly increased in the cortex of YAP^f/f^ ALS mice, suggesting the Wnt/β-catenin pathway was activated in YAP^f/f^ ALS mice. However, activation of the Wnt/β-catenin pathway was significantly decreased in the cortex of YAP^GFAP^-CKO ALS mice (Figure 5J-K). Double immunostaining further confirmed a significant reduction of EAAT2 expression in astrocytes of the motor cortex of YAP^GFAP^-CKO ALS mice (Figure 5L-M). Taken together, these results suggest that astrocytic EAAT2 expression is downregulated in the motor cortex of YAP^GFAP^-CKO ALS mice through downregulating the β-catenin signaling.

### Impaired glutamate uptake in YAP^-/-^ astrocytes exacerbates glutamate excitotoxicity in neuronal cells in ALS *in vitro* model

**Figure 6:**
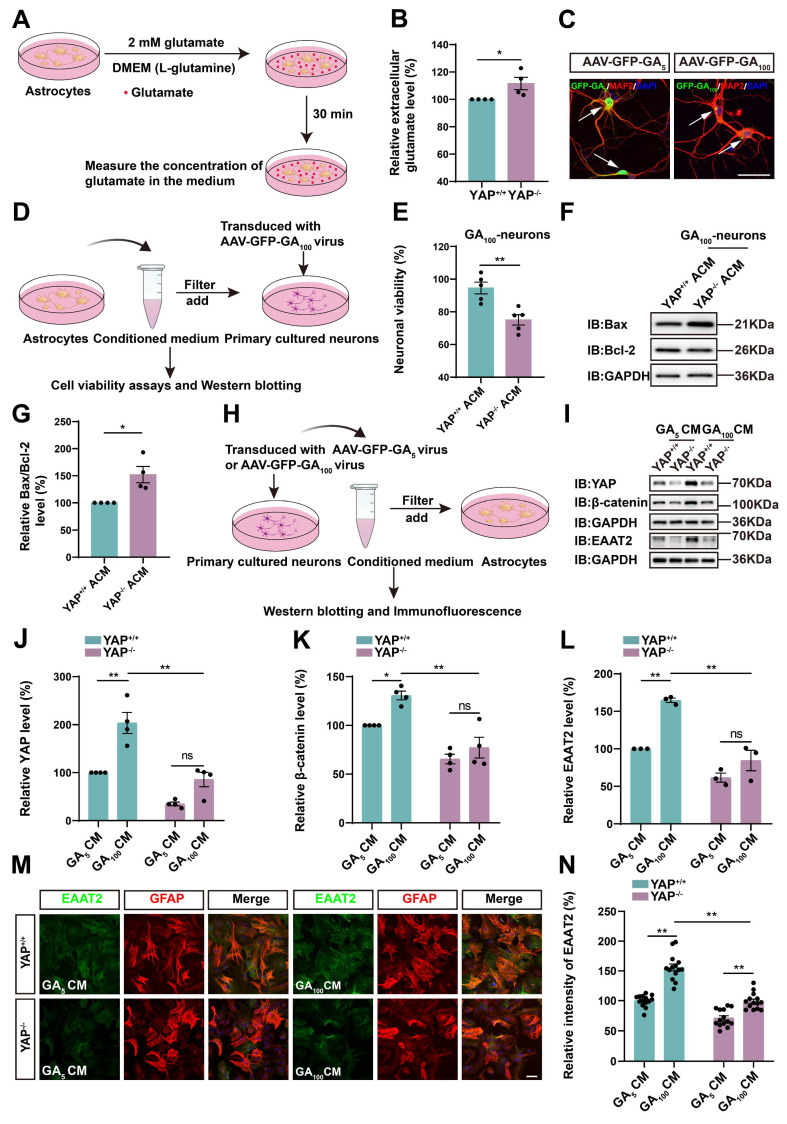
** Impaired glutamate uptake in YAP^-/-^ astrocytes exacerbates glutamate excitotoxicity in primary cultured neurons and the YAP/β-catenin/EAAT2 signaling pathway is activated in astrocytes in ALS *in vitro* model*.*** (**A**) Flowchart of the glutamate uptake assay. (**B**) Quantitative analysis of the relative extracellular glutamate levels in YAP^+/+^ and YAP^-/-^ astrocytes (n = 4 each group, Student's *t*-test). (**C**) Representative images of immunostaining of primary cultured neurons transduced with AAV-GFP-GA_5_ virus or AAV-GFP-GA_100_ virus. The arrows indicated neurons that expressed GFP-GA_5_ or GFP-GA_100_. GFP-GA_5_ and GFP-GA_100_, green; MAP2, red; DAPI, blue. (**D**) Flowchart of conditioned medium collected from YAP^+/+^ (YAP^+/+^ ACM) and YAP^-/-^ (YAP^-/-^ ACM) astrocytes and applied to primary cultured neurons transduced with AAV-GFP-GA_100_ virus for relevant experiments. (**E**) Neuronal viability was assessed by CCK-8 assay in primary cultured neurons transduced with AAV-GFP-GA_100_ virus and treated with YAP^+/+^ ACM or YAP^-/-^ ACM (n = 5 each group, Student's *t*-test). (**F**) Western blot analysis of Bcl-2 and Bax expression in primary cultured neurons transduced with AAV-GFP-GA_100_ virus and treated with YAP^+/+^ ACM or YAP^-/-^ ACM. (**G**) Quantitative analysis of the relative Bax/Bcl-2 expression level in primary cultured neurons as shown in (**F**) (n = 4 each group, normalized to GA_100_-YAP^+/+^ACM, Student's *t*-test). (**H**) Flowchart of conditioned medium collected from primary cultured neurons transduced with AAV-GFP-GA_5_ virus (GA_5_ CM) or AAV-GFP-GA_100_ virus (GA_100_ CM) and applied to YAP^+/+^ and YAP^-/-^ astrocytes for relevant experiments. (**I**) Western blot analysis of YAP, β-catenin and EAAT2 expression in YAP^+/+^ and YAP^-/-^ astrocytes treated with GA_5_ CM or GA_100_ CM. (**J-L**) Quantitative analysis of the relative YAP (**J**, n = 4 each group, normalized to YAP^+/+^-GA_5_ CM), β-catenin (**K**, n = 4 each group, normalized to YAP^+/+^-GA_5_ CM**)** and EAAT2 (**L**, n = 3 each group, normalized to YAP^+/+^-GA_5_ CM) expression levels in astrocytes as shown in (**I**). (**M**) Double immunostaining of EAAT2 (green) and GFAP (red) in YAP^+/+^ and YAP^-/-^ astrocytes treated with GA_5_ CM or GA_100_ CM. (**N**) Quantitative analysis of the relative intensity of EAAT2 as shown in (**M**) (n = 14 each group). Scale bars, 50 µm. Data were presented as mean ± SEM. Two-way ANOVA with Tukey's multiple comparisons test unless otherwise indicated, n.s., not significant (p > 0.05),^*^p < 0.05, ^**^p < 0.01.

We next examined whether impaired glutamate uptake in YAP-deficient astrocytes resulted in neuronal death under the C9orf72-poly-GA model *in vitro*. GFP-GA_5_ (Control) and GFP-GA_100_ plasmids were transfected into N2a cells (mouse neuroblastoma cell line) to mimic the C9orf72-poly-GA model *in vitro*. We first assessed whether YAP knockout affected glutamate uptake in astrocytes by measuring extracellular glutamate concentration using a glutamate uptake assay (Figure 6A). As expected, the results showed that extracellular glutamate concentration was significantly higher in YAP^-/-^ astrocytes (Figure 6B), suggesting impaired glutamate uptake in YAP^-/-^ astrocytes. We speculated that this was due to reduced EAAT2 expression in YAP^-/-^ astrocytes. To examine whether increased glutamate concentration caused toxic effects on neurons, we collected conditioned medium (CM) from YAP^+/+^ and YAP^-/-^ astrocytes, described as YAP^+/+^ ACM and YAP^-/-^ ACM, respectively, and applied it to N2a cells transfected with GFP-GA_5_ plasmid or GFP-GA_100_ plasmid (Supplementary Figure S3A). Flow cytometry analysis using Annexin V-APC/PI showed that the percentage of early and late apoptotic cells was significantly increased in N2a-GFP-GA_100_ cells treated with YAP^-/-^ ACM (Supplementary Figure S3B-C). Furthermore, western blot analysis showed that the ratio of anti-apoptotic protein Bcl-2/pro-apoptotic protein Bax was significantly downregulated in N2a-GFP-GA_100_ cells treated with YAP^-/-^ ACM (Supplementary Figure S3D-E).

In addition, we cultured primary neurons from the cortex and hippocampus of WT mice. AAV-GFP-GA_5_ (control) virus and AAV-GFP-GA_100_ virus transduced these neurons to mimic the ALS model *in vitro* (Figure 6C). Similarly, we collected conditioned medium (CM) from YAP^+/+^ (YAP^+/+^ ACM) and YAP^-/-^ (YAP^-/-^ ACM) astrocytes to treat primary cultured neurons transduced with AAV-GFP-GA_100_ virus (Figure 6D). As shown in Figure 6E, we found that the viability of neurons transduced with AAV-GFP-GA_100_ was significantly decreased after treatment with YAP^-/-^ ACM. Western blot results further confirmed the ratio of pro-apoptotic protein Bax/anti-apoptotic protein Bcl-2 was significantly increased in these neurons (Figure 6F-G). Collectively, these results suggest that impaired glutamate uptake in YAP^-/-^ astrocytes contributes to increased neuronal apoptosis following glutamate excitotoxicity under the pathology of C9orf72-ALS.

### Wnts secreted by degenerating neurons and/or astrocytes activate the YAP/β-catenin/EAAT2 signaling pathway in astrocytes

To further investigate the molecular mechanism of astrocytic YAP in C9orf72-poly-GA ALS, we collected CM from N2a cells transfected with GFP-GA_5_ plasmid and GFP-GA_100_ plasmid, described as GA_5_ CM and GA_100_ CM, respectively, and treated YAP^+/+^ and YAP^-/-^ astrocytes with them (Supplementary Figure S3F). Immunostaining showed that YAP was upregulated and translocated to the nucleus in YAP^+/+^ astrocytes treated with GA_100_ CM (Supplementary Figure S3G). We speculated that GFP-GA_100_-transfected N2a cells (degenerating neuronal cells), might secrete specific factors to activate YAP in astrocytes. Furthermore, western blot analysis demonstrated that the expression of β-catenin was upregulated in YAP^+/+^ astrocytes treated with GA_100_ CM. In contrast, the expression of β-catenin was significantly downregulated in YAP^-/-^ astrocytes treated with GA_100_ CM (Supplementary Figure S3H-I). Immunostaining further showed that the intensity of EAAT2 was significantly downregulated in YAP^-/-^ astrocytes treated with GA_100_ CM (Supplementary Figure S3J-K).

In addition, we collected CM from primary cultured neurons transduced with AAV-GFP-GA_5_ virus (GA_5_ CM) and AAV-GFP-GA_100_ virus (GA_100_ CM) to treat YAP^+/+^ and YAP^-/-^ astrocytes (Figure 6H). As shown in Figure 6I-L, the expression of YAP, EAAT2 and β-catenin were upregulated in YAP^+/+^ astrocytes treated with GA_100_ CM, however, GA_100_ CM failed to induce the upregulation of YAP, EAAT2 and β-catenin in YAP^-/-^ astrocytes. Immunostaining further confirmed that the upregulation of EAAT2 induced by GA_100_ CM was significantly reduced in YAP^-/-^ astrocytes (Figure 6M-N). These results suggest that degenerating neurons may secrete factors that activate YAP/β-catenin/EAAT2 signaling in WT astrocytes.

**Figure 7:**
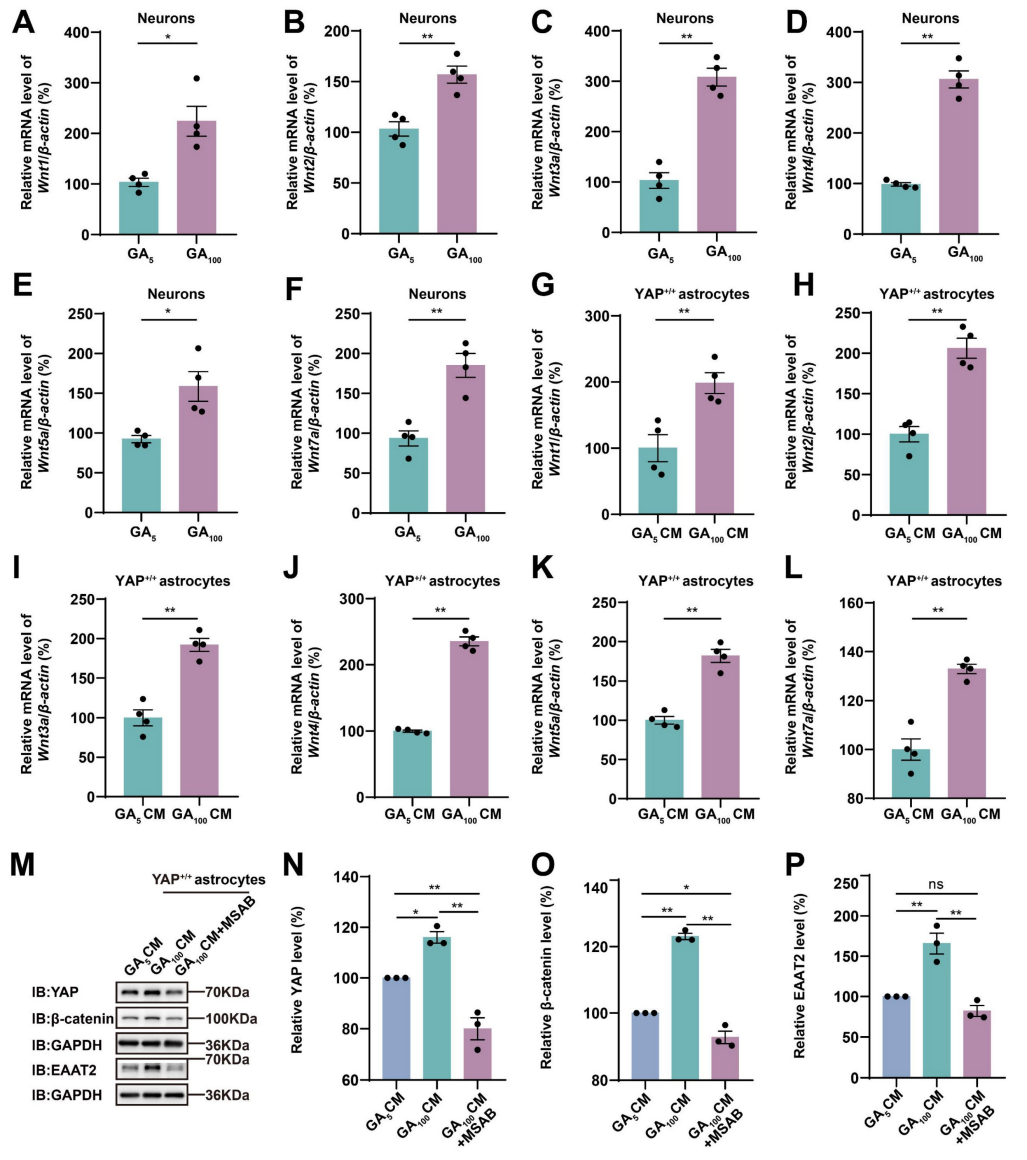
** Wnts secreted by degenerating neurons transfected with GFP-GA_100_ and/or astrocytes activate the YAP/β-catenin/EAAT2 signaling pathway in astrocytes.** (**A-F**) qPCR analysis of the relative mRNA levels of *Wnt1* (**A**), *Wnt2* (**B**), *Wnt3a* (**C**), *Wnt4* (**D**), *Wnt5a* (**E**) and *Wnt7a* (**F**) in primary cultured neurons transduced with AAV-GFP-GA_5_ virus or AAV-GFP-GA_100_ virus (n = 4 each group, Student's *t*-test). (**G-L**) qPCR analysis of the relative mRNA levels of *Wnt1* (**G**), *Wnt2* (**H**), *Wnt3a* (**I**), *Wnt4* (**J**), *Wnt5a* (**K**) and *Wnt7a* (**L**) in YAP^+/+^ astrocytes treated with GA_5_ CM or GA_100_ CM (n = 4 each group, Student's *t*-test). (**M**) Western blot analysis detected YAP, β-catenin and EAAT2 expression in YAP^+/+^ astrocytes 20 h after treatment with GA_5_ CM, GA_100_ CM or GA_100_ CM supplemented with 10 µM MSAB. (**N-P**) Quantitative analysis of the relative YAP (**N**), β-catenin (**O**) and EAAT2 (**P**) expression levels in YAP^+/+^ astrocytes as shown in (**M**) (n = 3 each group, normalized to YAP^+/+^-GA_5_ CM). Data were presented as mean ± SEM. One-way ANOVA with Tukey's multiple comparisons test unless otherwise indicated, n.s., not significant (*p > 0.05*),^*^p < 0.05,^**^p < 0.01.

The Wnt signaling pathway plays an important role in the physiological and pathophysiological processes of ALS 38, and interacts with Hippo/YAP signaling 43. Therefore, we next examined whether N2a cells transfected with GFP-GA_100_ could secrete Wnt-related ligands. qPCR results showed that *Wnt1*, *Wnt3a*, *Wnt4*, and *Wnt7a* mRNA were significantly upregulated in N2a cells transfected with GFP-GA_100_ (Supplementary Figure S3L, N-O, Q), but no significant differences were observed in *Wnt2* and *Wnt5a* mRNA (Supplementary Figure S3M, P). Furthermore, in primary cultured neurons transduced with AAV-GFP-GA_5_ virus or AAV-GFP-GA_100_ virus, we found that *Wnt1*, *Wnt2*, *Wnt3a*, *Wnt4*, *Wnt5a*, and *Wnt7a* mRNA were significantly upregulated in the latter (Figure 7A-F). Multiple studies have shown activation of the Wnt/β-catenin pathway in astrocytes under ALS conditions 39-41. Therefore, we treated YAP^+/+^ astrocytes with CM collected from primary cultured neurons transduced with AAV-GFP-GA_5_ virus or AAV-GFP-GA_100_ virus. qPCR results showed that *Wnt1*,* Wnt2*, *Wnt3a*,* Wnt4*, *Wnt5a*, and *Wnt7a* mRNA were significantly upregulated in YAP^+/+^ astrocytes treated with GA_100_ CM (Figure 7G-L). In addition, to determine whether GA_100_ CM induced upregulation of EAAT2 was through the Wnt/β-catenin pathway, we used MSAB, a potent and selective inhibitor of the Wnt/β-catenin pathway. Interestingly, the upregulation of YAP, β-catenin and EAAT2 expression induced by GA_100_ CM was significantly blocked by MSAB (Figure 7M-P). These results suggest that degenerating neurons and/or astrocytes secrete Wnts, such as *Wnt1* and/or *Wnt3a* to activate the YAP/β-catenin/EAAT2 signaling pathway in astrocytes. Taken together, these results indicate that impaired glutamate uptake in YAP^-/-^ astrocytes exacerbates glutamate excitotoxicity in neuronal cells in ALS by downregulating the astrocytic Wnt/YAP/β-catenin/EAAT2 signaling pathway *in vitro*.

### Activation of EAAT2 partially restores motor deficits and neuronal loss in YAP^GFAP^-CKO ALS mice

**Figure 8:**
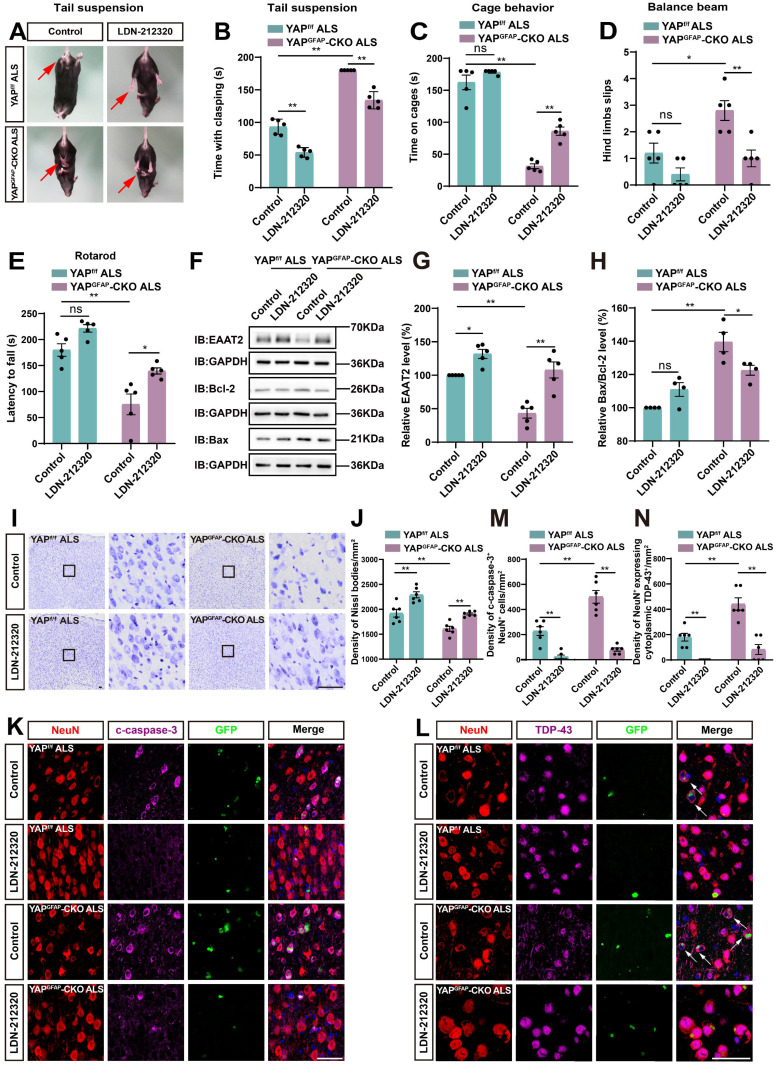
** Activation of EAAT2 partially restores motor deficits and neuronal loss in YAP^GFAP^-CKO ALS mice.** (**A**) Representative images of YAP^f/f^ ALS mice and YAP^GFAP^-CKO ALS mice treated with or without LDN-212320 treatment in tail-suspension test. The red arrows indicate the hind limbs of mice. (**B**) Quantitative analysis of hind limb clasping time of YAP^f/f^ ALS mice and YAP^GFAP^-CKO ALS mice treated with or without LDN-212320 treatment within 3 min in tail-suspension test (n = 5 each group). (**C**) Quantitative analysis of time keeping on the edges of cages of YAP^f/f^ ALS mice and YAP^GFAP^-CKO ALS mice treated with or without LDN-212320 treatment (n = 5 each group). (**D**) Quantitative analysis of the numbers of hind limb foot slips in the balance beam test of YAP^f/f^ ALS mice and YAP^GFAP^-CKO ALS mice treated with or without LDN-212320 (n = 5 each group). (**E**) Quantitative analysis of the latency to fall from the accelerated rotating rod of YAP^f/f^ ALS mice and YAP^GFAP^-CKO ALS mice treated with or without LDN-212320 (n = 5 each group). (**F**) Western blot analysis of EAAT2, Bax and Bcl-2 expression in the cortex of YAP^f/f^ ALS mice and YAP^GFAP^-CKO ALS mice treated with or without LDN-212320. (**G**) Quantitative analysis of the relative EAAT2 expression level as shown in (**F**) (n = 5 each group, normalized to control YAP^f/f^ ALS mice). (**H**) Quantitative analysis of the relative Bax/Bcl-2 expression level as shown in (**F**) (n = 4 each group, normalized to control YAP^f/f^ ALS mice). (**I**) Representative staining images of Nissl staining in the motor cortex of YAP^f/f^ ALS mice and YAP^GFAP^-CKO ALS mice treated with or without LDN-212320. (**J**) Quantitative analysis of the density of Nissl bodies as shown in (**I**) (n = 6 each group). (**K-L**) Double immunostaining of NeuN (red) and c-caspase-3 (far-red) (**K**), and NeuN (red) and TDP-43 (far-red) (**L**) in the motor cortex of YAP^f/f^ ALS mice and YAP^GFAP^-CKO ALS mice treated with or without LDN-212320. White arrows indicate cytoplasmic TDP-43 expression. (**M-N**) Quantitative analysis of the density of c-caspase-3^+^ cells in NeuN^+^ neurons (**M**) and the density of cytoplasmic TDP-43 in NeuN^+^ neurons (**N**) as shown in (**K**) and (**L**), respectively (n = 6 each group). Scale bars, 50 µm. Data were presented as mean ± SEM. Two-way ANOVA with Tukey's multiple comparisons test, n.s., not significant (*p > 0.05*),^*^p < 0.05,^**^p < 0.01.

EAAT2 can be upregulated through transcriptional or translational activation, and previous studies have also shown that treatment with LDN/OSU-212320 (an agonist of EAAT2) restores EAAT2 protein levels, enhances motor function, and prolongs lifespan in SOD1^G93A^ mice 71. To investigate whether EAAT2 activation restored motor deficits and neuronal loss in YAP^GFAP^-CKO ALS mice, we performed several experiments. As expected, the tail-suspension test showed that the hind limb clasping time of YAP^GFAP^-CKO ALS mice was significantly reduced after LDN-212320 treatment (Figure 8A-B). Meanwhile, the cage behavior test further showed that the walking time along the edge of the cage was significantly prolonged in LDN-212320-treated YAP^GFAP^-CKO ALS mice (Figure 8C). The balance beam test showed that LDN-212320-treated YAP^GFAP^-CKO ALS mice had fewer hind limb slips on the balance beam (Figure 8D), and the rotarod test showed that the latency to fall from the rotating rod was prolonged in LDN-212320-treated YAP^GFAP^-CKO ALS mice (Figure 8E). Collectively, these results suggest that activation of EAAT2 by LDN-212320 treatment partially restores behavioral deficits in the motor coordination and balance in YAP^GFAP^-CKO ALS mice.

Next, western blot analysis showed that after treatment with LDN-212320, the level of EAAT2 protein was significantly upregulated (Figure 8F-G), and the ratio of Bax/Bcl-2 was significantly decreased in YAP^GFAP^-CKO ALS mice (Figure 8F, H). In addition, Nissl staining showed that neuronal loss was significantly inhibited in the motor cortex of LDN-212320-treated YAP^GFAP^-CKO ALS mice (Figure 8I-J). Double immunostaining for NeuN and c-caspase-3 showed that LDN-212320 treatment significantly reduced the density of c-caspase-3 positive neurons in the motor cortex of YAP^GFAP^-CKO ALS mice (Figure 8K, M). We then examined the translocation of TDP-43 in the motor cortex of control and LDN-212320-treated mice. Interestingly, double immunostaining showed that pathological translocation of TDP-43 was significantly reduced in LDN-212320-treated YAP^GFAP^-CKO ALS mice, and TDP-43 was primarily expressed in the nucleus of neurons (Figure 8L, N). Taken together, these results suggest that activation of EAAT2 partially restores motor deficits and neuronal loss in YAP^GFAP^-CKO ALS mice.

### Activation of astrocytic YAP/β-catenin/EAAT2 signaling alleviates motor deficits and neurodegeneration in C9orf72-poly-GA mice

**Figure 9:**
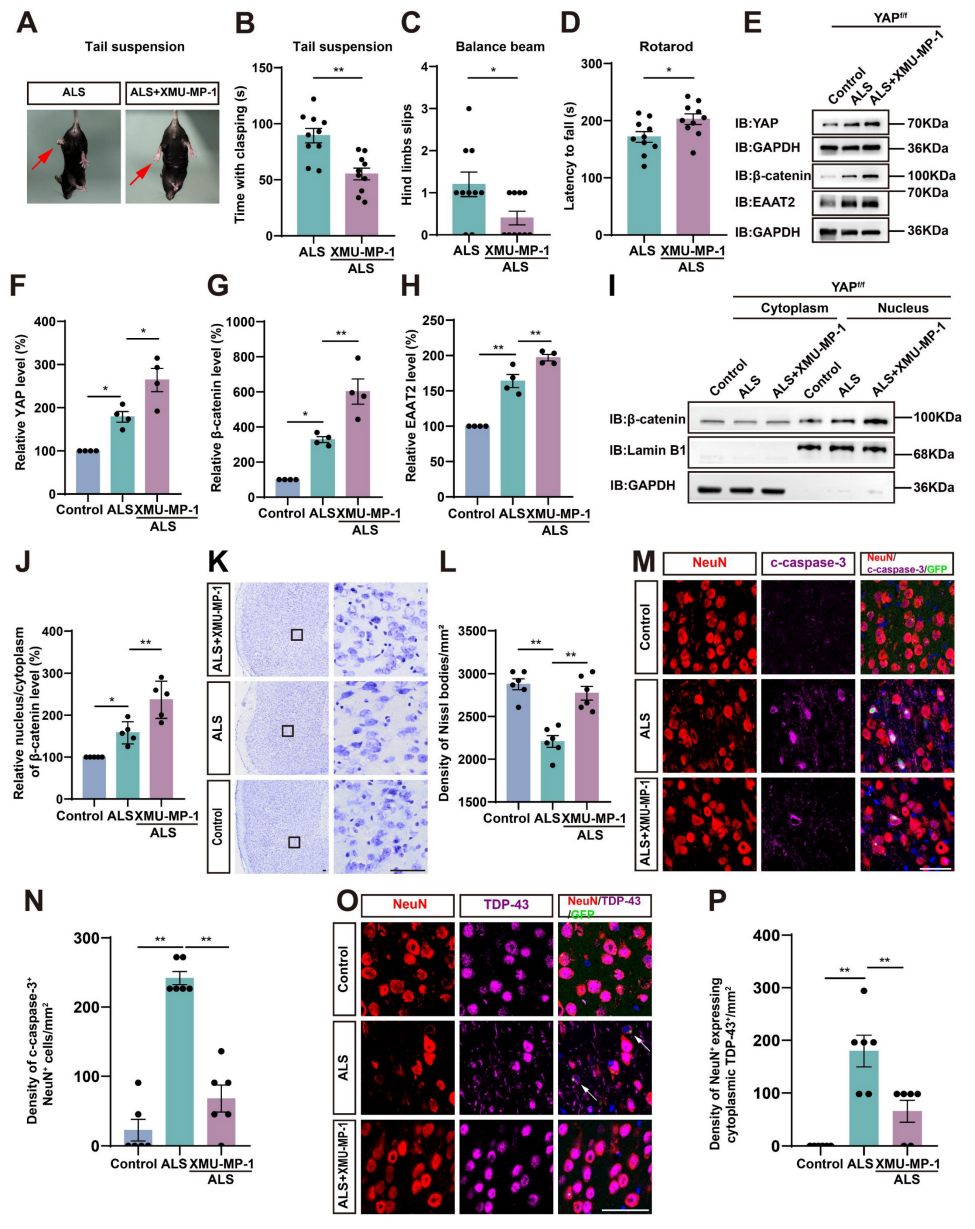
** Activation of astrocytic YAP/β-catenin/EAAT2 signaling alleviates motor deficits and neurodegeneration in C9orf72-poly-GA mice.** (**A**) Representative images of ALS mice and XMU-MP-1-treated ALS mice in tail-suspension test. The red arrows indicate the hind limbs of mice. (**B**) Quantitative analysis of hind limb clasping time of ALS mice and XMU-MP-1-treated ALS mice within 3 min in tail-suspension test (n = 10 each group, Student's *t*-test). (**C**) Quantitative analysis of the numbers of hind limb foot slips in the balance beam test of ALS mice and XMU-MP-1-treated ALS mice (n = 10 each group, Student's *t*-test). (**D**) Quantitative analysis of latencies to fall from the accelerated rotating beams of ALS mice and XMU-MP-1-treated ALS mice (n = 10 each group, Student's *t*-test). (**E**) Western blot analysis of YAP, β-catenin and EAAT2 expression in the cortex of control mice, ALS mice and XMU-MP-1-treated ALS mice. (**F-H**) Quantitative analysis of the relative YAP (**F**), β-catenin (**G**) and EAAT2 (**H**) expression levels as shown in (**E**) (n = 4 each group, normalized to control mice). (**I**) Western blot analysis of cytoplasmic and nuclear expression of β-catenin in the cortex of control mice, ALS mice, and XMU-MP-1-treated ALS mice. (**J**) Quantitative analysis of the relative nucleus/cytoplasm ratio of β-catenin expression level as shown in (**I**) (n = 5 each group, normalized to control mice). (**K**) Representative staining images of Nissl staining in the motor cortex of control mice, ALS mice and XMU-MP-1-treated ALS mice. (**L**) Quantitative analysis of the density of Nissl bodies as shown in (**I**) (n = 6 each group). (**M, O**) Double immunostaining of NeuN (red) and c-caspase-3 (far-red) (**M**), and NeuN (red) and TDP-43 (far-red) (**O**) in the motor cortex of control mice, ALS mice and XMU-MP-1-treated ALS mice. White arrows indicate cytoplasmic TDP-43 expression. (**N, P**) Quantitative analysis of the density of c-caspase-3^+^ cells in NeuN^+^ neurons (**N**) and the density of cytoplasmic TDP-43 in NeuN^+^ neurons (**P**) as shown in (**M**) and (**O**), respectively (n = 6 each group). Scale bars, 50 µm. Data were presented as mean ± SEM. One-way ANOVA with Tukey's multiple comparisons test unless otherwise indicated,^*^p < 0.05,^**^p < 0.01.

To investigate whether activation of astrocytic YAP could alleviate the symptoms of C9orf72-poly-GA mice by upregulating EAAT2, we treated ALS mice with XMU-MP-1, a compound that activates the downstream effector YAP by blocking MST1/2 kinase activity 72. Interestingly, the tail-suspension test showed that the hind limb clasping time was significantly reduced in C9orf72-poly-GA mice after XMU-MP-1 treatment (Figure 9A-B). The balance beam test further showed that the number of hind limb slips were significantly decreased in XMU-MP-1-treated C9orf72-poly-GA mice (Figure 9C), and the rotarod test showed that the latency to fall from the rotating rod was prolonged in XMU-MP-1-treated C9orf72-poly-GA mice (Figure 9D). These results indicate that XMU-MP-1 improves the recovery of motor function in C9orf72-poly-GA mice.

As expected, western blot analysis further showed that YAP signaling was activated in the cortex of C9orf72-poly-GA mice treated with XMU-MP-1(Figure 9E-F), with significant increases in β-catenin and EAAT2 protein levels (Figure 9E, G-H). In addition, cytosol-nuclei fractionation experiments showed that XMU-MP-1 treatment increased the nucleus/cytoplasm ratio of β-catenin in the cortex of C9orf72-poly-GA mice (Figure 9I-J), indicating that the Wnt/β-catenin pathway was activated in these mice. Nissl staining showed that the loss of Nissl bodies in the motor cortex was significantly reduced in XMU-MP-1-treated C9orf72-poly-GA mice (Figure 9K-L). Double immunostaining for NeuN and c-caspase-3 further showed that neuronal death in the motor cortex of C9orf72-poly-GA mice was significantly reduced after XMU-MP-1 treatment (Figure 9M-N). Moreover, double immunostaining for TDP-43 and NeuN showed that TDP-43 was primarily localized in the nucleus of neurons in the motor cortex of C9orf72-poly-GA mice treated with XMU-MP-1 (Figure 9O-P). Taken together, these results suggest that activation of astrocytic YAP/β-catenin /EAAT2 signaling alleviates motor deficits and neurodegeneration in C9orf72-poly-GA mice.

## Discussion

**Figure 10:**
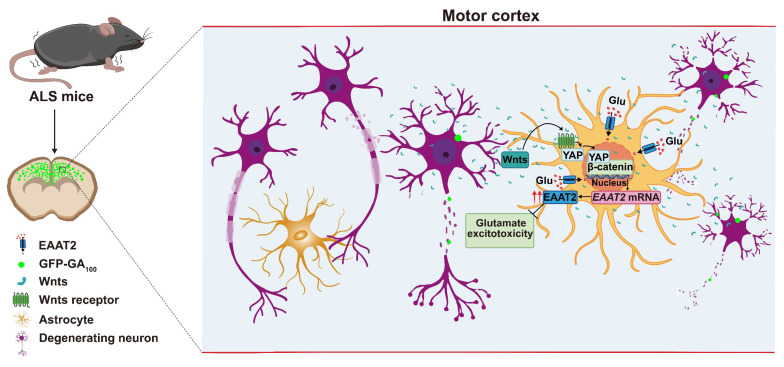
** A working model of astrocytic YAP's function in C9orf72-poly-GA mice.** In the motor cortex of C9orf72-poly-GA mice, degenerating neurons and/or astrocytes release the help signals by secreting factors such as Wnts to activate YAP/β-catenin signaling, and further upregulate EAAT2 expression in astrocytes. This cascade prevents glutamate excitotoxicity, neuronal loss, and motor dysfunction in C9orf72-poly-GA mice.

In this study, we provide evidence supporting the role and mechanism of astrocytic YAP-EAAT2 signaling in C9orf72-poly-GA mice and propose a working model (Figure 10). In this model, degenerating neurons and/or astrocytes in ALS mice may release the help signals by secreting factors such as Wnts, which activate the YAP/β-catenin signaling in astrocytes to upregulate EAAT2. This mechanism mitigates neuronal glutamate excitotoxicity, neuronal loss, and motor dysfunction in C9orf72-poly-GA mice. In addition, activation of YAP signaling upregulates β-catenin transcriptional activity and increases EAAT2 expression, thereby preventing neuronal loss and motor dysfunction in C9orf72-poly-GA mice. These findings reveal a previously unrecognized mechanism of self-protection in degenerating neurons mediated by astrocytic YAP through Wnts/β-catenin/EAAT2 signaling to prevent glutamate excitotoxicity of neurons in ALS mice, and provide a potential new drug target for ALS.

Recent studies have shown that YAP is not expressed in spinal cord neurons, but is upregulated and activated in astrocytes in a Hippo kinase-dependent manner, promoting the formation of glial scars and neural regeneration of mice after spinal cord injury 33. In EAE mice, astrocytic YAP also prevents the demyelination by inhibiting the upregulation and activation of Hippo pathway in astrocytes and promotes the expression of cholesterol synthesis gene in EAE 34. Consistent with these previous studies, this study showed that YAP was upregulated and activated in astrocytes in C9orf72-poly-GA mice, preventing motor deficits and neuronal loss by promoting the expression of EAAT2, suggesting that YAP in astrocytes may be activated and play conservatively protective roles in various disease models. Notably, a previous study has shown that YAP deltaCs (pro-survival isoforms of YAP) is progressively decreased in spinal cord neurons of SOD1^G93A^ transgenic ALS mice 73. These differing observations may be attributed to the use of different mouse models (C9orf72-poly-GA versus SOD1^G93A^) or the distinct brain regions examined (motor cortex versus spinal cord). In the future, it is interesting to test whether astrocytic YAP signaling is activated in a manner dependent on the inactivation of Hippo kinases in ALS.

By integrating the snRNA-seq of C9orf72-ALS/FTD patients with low and high YAP expression, and RNA-seq from cultured YAP^+/+^ and YAP^-/-^ astrocytes, we found that the most differential pathway was enriched in neurotransmitter uptake (Figure 5A-B). The MA-plot of YAP^+/+^ and YAP^-/-^ astrocytes showed that neurotransmitter uptake-related genes such as *Slc6a11*, *Slc1a2* (*EAAT2*), and *Slc1a3* (*EAAT1*) were significantly downregulated (Figure 5C). Indeed, glutamate excitotoxicity caused by dysfunction of EAAT2 is considered to play a crucial role in the pathogenesis of ALS 8, 70. Riluzole is a drug approved by the FDA for the treatment of ALS, which has been shown to inhibit excitotoxicity 74, 75. Previous studies have shown that glutamate transport is impaired in the brain and spinal cord of ALS patients 76. In addition, it has been reported that EAAT2 protein levels are decreased in the motor cortex and spinal cord of ALS 17, 77. However, some studies have also shown that EAAT2 expression was increased in the middle laminae of the motor cortex in patients 78, and EAAT2 was significantly upregulated in astrocytes induced by ALS patient-derived iPSC lines after co-culturing with neurons *in vitro* 79. In SOD1^G93A^ mice, no changes in EAAT2 expression were observed at 10 weeks of age; however, a progressive decrease in EAAT2 expression was observed in the ventral, but not in the dorsal, horn of the lumbar spinal cord at 14 and 18 weeks of age 80. In our study, we found that EAAT2 expression was increased in 3-month-old C9orf72-poly-GA mice and decreased in YAP^GFAP^-CKO ALS mice. This discrepancy may potentially be explained by the different roles of EAAT2 in the disease progression of ALS.

Several lines of evidence suggest that glutamate excitotoxicity in ALS is regulated by astrocytic YAP-EAAT2 signaling. Firstly, the extracellular glutamate concentration was significantly higher in YAP^-/-^ astrocytes (Figure 6B). Secondly, the apoptosis of primary cultured neurons transduced with AAV-GFP-GA_100_ (Figure 6E-G) or N2a cells transfected with GFP-GA_100_ (Supplementary Figure 3B-E) was aggravated after treatment with conditioned medium from YAP^-/-^ astrocytes. Thirdly, treatment with LDN-212320, an agonist of EAAT2, promoted EAAT2 expression and partially restored motor deficits and neuronal loss in YAP^GFAP^-CKO ALS mice (Figure 8). Finally, activation of YAP by XMU-MP-1 upregulated β-catenin and EAAT2 expression, which partially alleviated neurodegeneration and motor deficits in ALS mice (Figure 9). How is YAP/EAAT2 signaling activated in ALS mice? Our recent studies have shown that YAP signaling in astrocytes promotes EAAT2 expression by regulating the transcriptional activity of β-catenin 54. Consistent with these findings, in our studies, we found that β-catenin was downregulated in YAP^GFAP^-CKO ALS mice and YAP^-/-^ astrocytes, and upregulated and translocated into the nucleus by the activation of YAP signaling. Interestingly, YAP/EAAT2 signaling was activated by conditioned medium from N2a cells transfected with GFP-GA_100_ or primary cultured degenerating neurons, suggesting that degenerating neurons with GA aggregates might secrete factors that activate YAP/β-catenin/EAAT2 signaling in astrocytes.

It has been reported that the expression of *Wnt1*, *Wnt2*, *Wnt3a* and *Wnt7a* is upregulated in SOD1^G93A^ mice, and in ALS, motor neurons, astrocytes, microglia and oligodendrocytes exhibit high levels of Wnt/β-catenin signaling and β-catenin activation 37, 38. The Wnt/β-catenin signaling pathway activated by neurodegeneration is related to the proliferation of spinal glial cells in SOD1^G93A^ transgenic mice 40. In fact, previous studies have shown that Wnts such as *Wnt1* and *Wnt3a* are upregulated in astrocytes in ALS 81. In this study, primary cultured neurons were transduced with AAV-GFP-GA_5_ and AAV-GFP-GA_100_ viruses to establish an *in vitro* ALS model (Figure 6C). qPCR results showed that *Wnt1*, *Wnt2*,* Wnt3a*,* Wnt4*,* Wnt5a*, and *Wnt7a* mRNA were significantly upregulated in primary cultured neurons transduced with AAV-GFP-GA_100_ virus (Figure 7A-F), suggesting that degenerating neurons upregulated *Wnts* mRNA, consistent with the results in N2a cell line. Meanwhile, as shown in Figure 7G-L, we found that *Wnt1*,* Wnt2*,* Wnt3a*,* Wnt4*,* Wnt5a*, and* Wnt7a* mRNA were significantly upregulated in YAP^+/+^ astrocytes treated with conditioned medium from primary cultured neurons transduced with AAV-GFP-GA_100_ virus, suggesting that degenerating neurons somehow promoted the upregulation expression of Wnts in astrocytes. Thus, astrocytes both receive Wnt signals and express Wnts in ALS. Interestingly, in the MPTP mouse model of Parkinson's disease, previous studies documented the early up-regulation of astrocytic Wnt1 in the ventral midbrain of degenerating substantia nigra neurons as a self-repair intersystem neuroprotective crosstalk 47-50. Based on these previous studies and our results (Figure 7 and Supplementary Figure S3), it is possible that early in ALS pathogenesis, Wnts are secreted from neurons and/or astrocytes during neurodegeneration, then in turn activate the YAP/β-catenin/EAAT2 signaling pathway in astrocytes. However, YAP may regulate EAAT2 expression through its interaction with β-catenin, and the precise mechanism underlying this process requires further investigation in ALS. Moreover, we also observed an intriguing phenomenon in which GFP-GA_100_ aggregates increased significantly in the cortex of YAP^GFAP^-CKO ALS mice. We hypothesize that an additional mechanism may be influencing the C9orf72-poly-GA mice, and we plan to further explore the mechanisms involved in future studies.

Riluzole has been shown to reduce the release of glutamate from nerve endings 74, 75. Interestingly, Riluzole treatment upregulates the Wnt3a expression in melanoma cells *in vitro,* and enhances the Wnt/β-catenin signaling in both HT22 neuronal cells and adult hippocampal progenitor cells 82, 83, suggesting that Riluzole is an enhancer of the Wnt/β-catenin signaling. Thus, it is interesting to test whether Riluzole also activates Wnt/β-catenin/YAP/EAAT2 signaling in astrocytes to perform neuroprotective effects in ALS in future. LDN/OSU-212320 can increase the expression of EAAT2 through translational activation, prevent excitotoxicity, delay the decline of motor function, and prolong the lifespan of SOD1^G93A^ mice 71. Consequently, increasing the expression of EAAT2 could serve as a potential treatment to prevent excitotoxicity in neurodegenerative diseases. Consistent with the previous results, treatment with LDN-212320 upregulated the expression of EAAT2 in the motor cortex of YAP^GFAP^-CKO ALS mice, preventing neuronal loss and motor deficits (Figure 8). XMU-MP-1 (an inhibitor of Hippo kinase MST1/2) is used to activate YAP signaling 72. Our results showed that XMU-MP-1 activated YAP signaling and partially improved motor function recovery in C9orf72-poly-GA mice (Figure 9). These results suggest that the Hippo/YAP pathway plays a critical role in regulating the glutamate transporter EAAT2 and maintaining glutamate homeostasis in astrocytes. Therefore, it will be interesting to test the combination of these two drugs (LDN-212320 or Riluzole with XMU-MP-1) for potential synergistic therapeutic effects in ALS.

In summary, our studies identify an unrecognized mechanism of self-protection in degenerating neurons, mediated by astrocytic YAP through Wnt/β-catenin/EAAT2 signaling, which prevents glutamate excitotoxicity in neurons of ALS mice. This highlights the therapeutic potential of the YAP/Wnt/β-catenin/EAAT2 signaling pathway in delaying and treating ALS diseases, which may contribute to the development of new therapeutic methods for ALS.

## Supplementary Material

Supplementary figures.

## Abbreviations

ALS: Amyotrophic lateral sclerosis
CNS: central nervous system
YAP: Yes-associated protein
CKO: conditional knockout
qPCR: quantitative real-time PCR
EAAT2: excitatory amino acid transporter-2
PBA: pseudobulbar affect
ASOs: antisense oligonucleotides
iPSCs: induced pluripotent stem cells
SOD1: Cu/Zn superoxide dismutase 1 gene
C9ORF72: Chromosome 9 Open Reading Frame 72
TARDBP: TAR DNA Binding Protein
FUS: Fused in Sarcoma
TBK1: TANK-binding kinase 1
MST1/2: mammalian Sterile 20-like kinases 1/2
SAV1: salvador homolog 1
MOB1A/B: Mps one binder 1A and B
LATS1/2: large tumor suppressor 1/2
TAZ: transcriptional co-activator with PDZ binding motif
TEAD: transcriptional enhanced associate domain
EAE: experimental autoimmune encephalomyelitis
MS-ON: multiple sclerosis-related optic neuritis
P0: postnatal day 0
PBS: phosphate buffered saline
PFA: paraformaldehyde
BSA: bovine serum albumin
TBST: tris buffered saline with Tween 20
N2a: Neuro-2a
FBS: fetal bovine serum
PS: penicillin/streptomycin
CM: conditioned medium
WT: wild-type
DIV: days *in vitro*
MOIs: multiplicities of infection
CCK-8: Cell Counting Kit-8
ACM: astrocyte conditioned medium
GFAP: glial fibrillary acidic protein
p-YAP: phosphorylated YAP
c-caspase-3: Cleaved-caspase-3
NF: neurofilament heavy polypeptide
snRNA-seq: single-nucleus transcriptomic
RNA-seq: RNA sequencing
GO: Gene Ontology
MA-plot: M-versus-A plot
