# Supplementary material for: An unrecognized mechanism of self-protection in degenerating neurons mediated by astrocytic YAP through Wnts/β-catenin/EAAT2 signaling in C9orf72-poly-GA mice
Source: Theranostics. 2025 Jul 24;15(16):8176–201. doi: 10.7150/thno.113599 (PMC12374586; doi:10.7150/thno.113599)
Supplement: Supplementary file 1 — Supplementary figures. [file thnov15p8176s1.pdf]

Supporting Information

An unrecognized mechanism of self-protection in degenerating neurons mediated by astrocytic YAP through Wnts/β-catenin/EAAT2 signaling in C9orf72-poly-GA mice

Dongmei Li[1#], Yan Wei[1#], Rui Yang[1#], Xuan Luo[1], Yanzhu Liu[1, 2], Weiqiao Zhao[3], Hui Yang[1, 2], Yumin Wu[4], Ying Wang[2*], Zhihui Huang[1*]

[1]School of Pharmacy, Hangzhou Normal University, Hangzhou 311121, Zhejiang, China.

[2]Department of Clinical Research Center, Affiliated Hangzhou First People's Hospital, Westlake University School of Medicine, Hangzhou 310006, Zhejiang, China.

[3]School of Basic Medicine, Hangzhou Normal University, Hangzhou 311121, Zhejiang, China.

[4]Department of Orthopedics (Spine Surgery), the First Affiliated Hospital of Wenzhou Medical University, Wenzhou 325000, Zhejiang, China.

[#]These authors contribute equally this work.

[*]Corresponding author:

Ying Wang, Email: nancywangying@163.com.

Zhihui Huang, Email: huang0069@hznu.edu.cn.

Supplementary Figure S1.

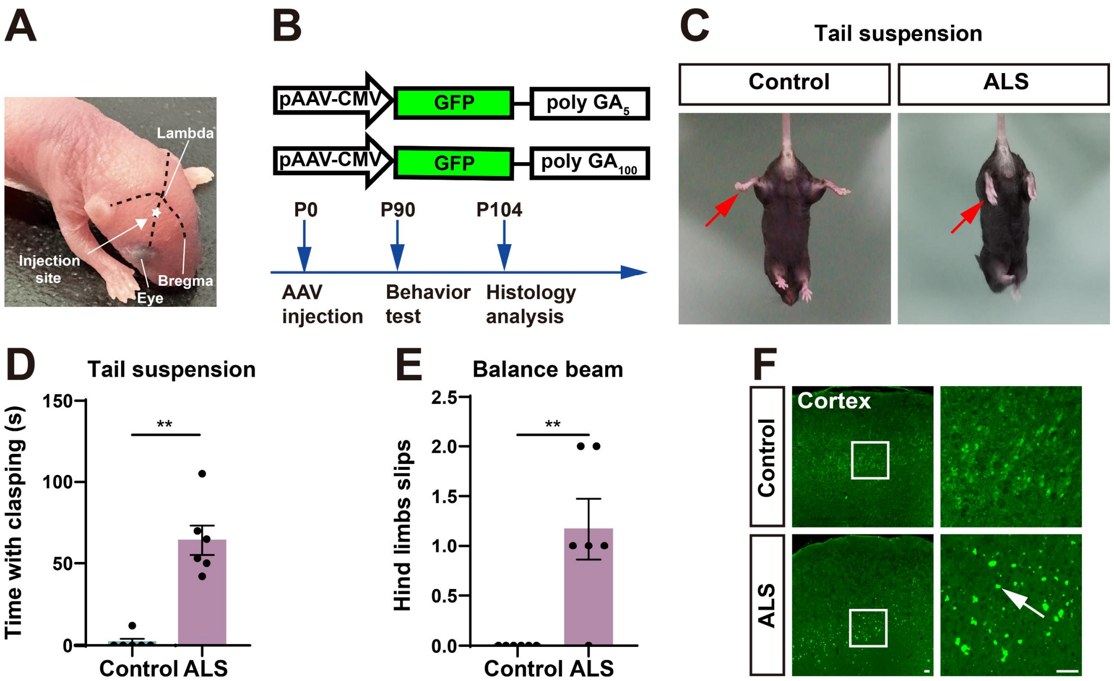

Supplementary Figure S1. Establishment of C9orf72-poly-GA ALS mice model. (**A**) Representative images of neonatal intraventricular AAV injection in mice. (**B**) Information of the AAV constructs and diagram of the experimental procedure. (**C**) Representative images of mice injected with AAV-GFP-GA_5 (control mice) and mice injected with AAV-GFP-GA_{100} (ALS mice) in tail-suspension test. The red arrows indicated the hind limbs of mice. (**D**) Quantitative analysis of hind limb clasping time of control and ALS mice within 3 min in tail-suspension test (n = 6 mice each group). (**E**) Quantitative analysis of the number of hind limb foot slips in the balance beam test in control and ALS mice (n = 6 mice each group). (**F**) Representative images of the cortex section of control and ALS mice. White arrow represented GFP-GA_{100} aggregates. Scale bars, 50 μm. Data were presented as mean ± SEM. Student's *t*-test, $^{**}p < 0.01$.

Supplementary Figure S2.

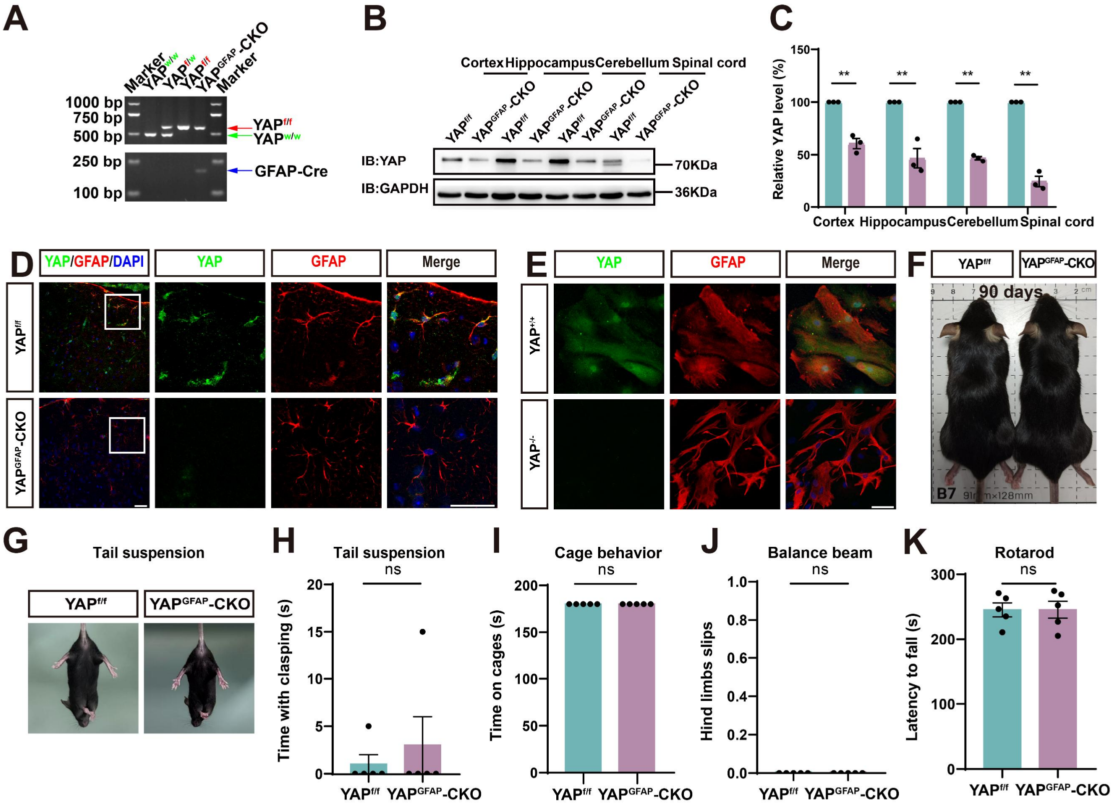

Supplementary Figure S2. Identification of YAP^{GFAP}-CKO mice and normal motor function in YAP^{GFAP}-CKO mice.

(**A**) Representative images of genotyping of YAP$^{f/f}$ and YAP^{GFAP}-CKO mice. (**B**) Western blot detected YAP expression in the cortex, hippocampus, cerebellum and spinal cord of 3-month-old YAP$^{f/f}$ and YAP^{GFAP}-CKO mice. (**C**) Quantitative analysis of YAP expression as shown in (**B**) (n = 3 each group, normalized to YAP$^{f/f}$ mice). (**D**) Double immunostaining analysis of YAP (green) and GFAP (red) in the motor cortex of 3-month-old YAP$^{f/f}$ and YAP^{GFAP}-CKO mice. (**E**) Double immunostaining analysis of YAP (green) and GFAP (red) in cultured YAP$^{+/+}$ and YAP$^{-/-}$ astrocytes. (**F**) Representative body size images of 3-month-old YAP$^{f/f}$ and YAP^{GFAP}-CKO mice. (**G**) Representative images of 3-month-old YAP$^{f/f}$ and YAP^{GFAP}-CKO mice in tail-suspension test. (**H**) Quantitative analysis of hind limb clasping time of YAP$^{f/f}$ and YAP^{GFAP}-CKO mice within 3 min in tail-suspension test (n = 5 mice each group). (**I**) Quantitative analysis of time keeping on the edges of cages in 3-month-old YAP$^{f/f}$ and YAP^{GFAP}-CKO mice (n = 5 mice each group). (**J**) Quantitative analysis of the number of hind limb foot slips in the balance beam test in 3-month-old YAP$^{f/f}$ and YAP^{GFAP}-

CKO mice (n = 5 mice each group). (**K**) Quantitative analysis of the latency to fall from the accelerated rotating rod of 3-month-old $YAP^{f/f}$ and YAP^{GFAP}-CKO mice (n = 5 each group). Scale bars, 50 μm. Data were presented as mean ± SEM. Student's *t*-test, n.s, not significant ($p > 0.05$), $^{**}p < 0.01$.

Supplementary Figure S3.

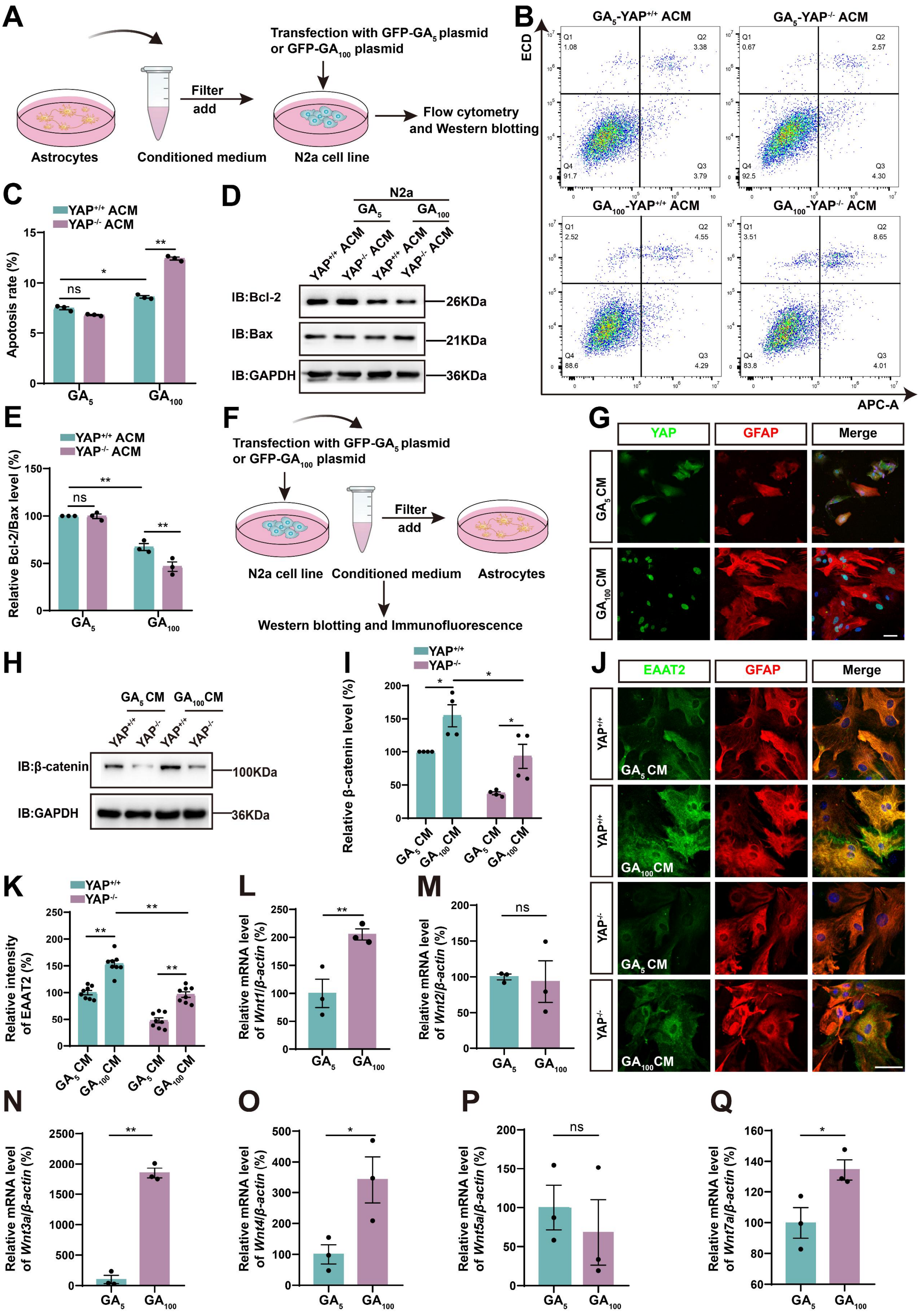

Supplementary Figure S3. Impaired glutamate uptake in YAP$^{-/-}$ astrocytes exacerbates glutamate excitotoxicity in neuronal cells transfected with GFP-GA$_{100}$, and Wnts secreted by degenerating neuronal cells transfected with GFP-GA$_{100}$

activate the YAP/β-catenin/EAAT2 signaling pathway in astrocytes.

(**A**) Flowchart of conditioned medium collected from $YAP^{+/+}$ ($YAP^{+/+}$ ACM) and $YAP^{-/-}$ ($YAP^{-/-}$ ACM) astrocytes and applied to N2a cells transfected with GFP-GA_5 plasmid or GFP-GA_{100} plasmid for relevant experiments. (**B**) Flow cytometry showed the apoptosis rate of N2a cells transfected with GFP-GA_5 plasmid or GFP-GA_{100} plasmid and treated with $YAP^{+/+}$ ACM or $YAP^{-/-}$ ACM. (**C**) Quantitative analysis of apoptosis rate of N2a cells, as shown in (**B**) ($n = 3$ each group, normalized to GA_5-$YAP^{+/+}$ ACM). (**D**) Western blot analysis of Bcl-2 and Bax expression in N2a cells transfected with GFP-GA_5 plasmid or GFP-GA_{100} plasmid and treated with $YAP^{+/+}$ ACM or $YAP^{-/-}$ ACM. (**E**) Quantitative analysis of the relative Bcl-2/Bax expression level in N2a cells as shown in (**D**) ($n = 3$ each group, normalized to GA_5-$YAP^{+/+}$ ACM). (**F**) Flowchart of conditioned medium collected from N2a cells transfected with GFP-GA_5 plasmid (GA_5 CM) or GFP-GA_{100} plasmid (GA_{100} CM) and applied to $YAP^{+/+}$ and $YAP^{-/-}$ astrocytes for relevant experiments. (**G**) Double immunostaining of YAP (green) and GFAP (red) in $YAP^{+/+}$ astrocytes treated with GA_5 CM or GA_{100} CM. (**H**) Western blot analysis of β-catenin expression in $YAP^{+/+}$ and $YAP^{-/-}$ astrocytes treated with GA_5 CM or GA_{100} CM. (**I**) Quantitative analysis of the relative β-catenin expression level in astrocytes as shown in (**H**) ($n = 4$ each group, normalized to $YAP^{+/+}$-GA_5 CM). (**J**) Double immunostaining of EAAT2 (green) and GFAP (red) in $YAP^{+/+}$ and $YAP^{-/-}$ astrocytes treated with GA_5 CM or GA_{100} CM. (**K**) Quantitative analysis of the relative intensity of EAAT2, as shown in (**J**) ($n = 8$ each group). (**L-Q**) qPCR analysis of the relative mRNA levels of *Wnt1* (**L**), *Wnt2* (**M**), *Wnt3a* (**N**), *Wnt4* (**O**), *Wnt5a* (**P**) and *Wnt7a* (**Q**) in N2a cells transfected with GFP-GA_5 plasmid or GFP-GA_{100} plasmid ($n = 3$ each group, Student's *t*-test). Scale bars, 50 μm. Data were presented as mean ± SEM. Two-way ANOVA with Tukey's multiple comparisons test unless otherwise indicated, n.s., not significant ($p > 0.05$), $^{*}p < 0.05$, $^{**}p < 0.01$.
